# SLAMM: Visual monocular SLAM with continuous mapping using multiple maps

**DOI:** 10.1371/journal.pone.0195878

**Published:** 2018-04-27

**Authors:** Hayyan Afeef Daoud, Aznul Qalid Md. Sabri, Chu Kiong Loo, Ali Mohammed Mansoor

**Affiliations:** Faculty of Computer Science and Information Technology, University of Malaya, Lembah Pantai, Kuala Lumpur, Malaysia; Institut de Robòtica i Informàtica Industrial, SPAIN

## Abstract

This paper presents the concept of Simultaneous Localization and Multi-Mapping (SLAMM). It is a system that ensures continuous mapping and information preservation despite failures in tracking due to corrupted frames or sensor’s malfunction; making it suitable for real-world applications. It works with single or multiple robots. In a single robot scenario the algorithm generates a new map at the time of tracking failure, and later it merges maps at the event of loop closure. Similarly, maps generated from multiple robots are merged without prior knowledge of their relative poses; which makes this algorithm flexible. The system works in real time at frame-rate speed. The proposed approach was tested on the KITTI and TUM RGB-D public datasets and it showed superior results compared to the state-of-the-arts in calibrated visual monocular keyframe-based SLAM. The mean tracking time is around 22 milliseconds. The initialization is twice as fast as it is in ORB-SLAM, and the retrieved map can reach up to 90 percent more in terms of information preservation depending on tracking loss and loop closure events. For the benefit of the community, the source code along with a framework to be run with Bebop drone are made available at https://github.com/hdaoud/ORBSLAMM.

## 1 Introduction

**Fig 1:**
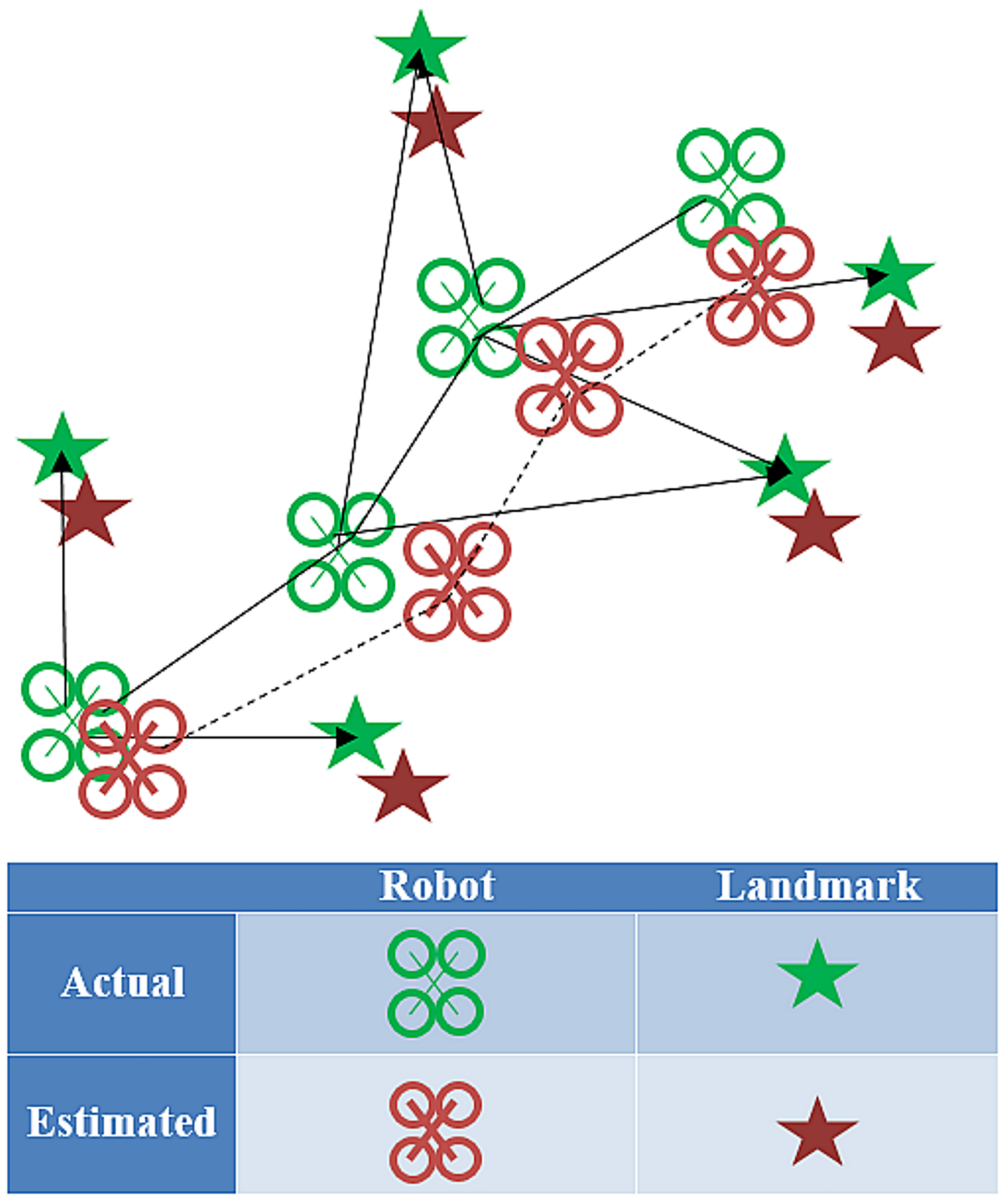
SLAM system.

Simultaneous Localization and Mapping (SLAM) is the problem of placing a robot at an unknown location in an unknown environment, then, using the onboard sensors, the robot would try to construct a map of the surroundings and utilize this map to localize itself (please see Fig 1)

**Fig 2:**
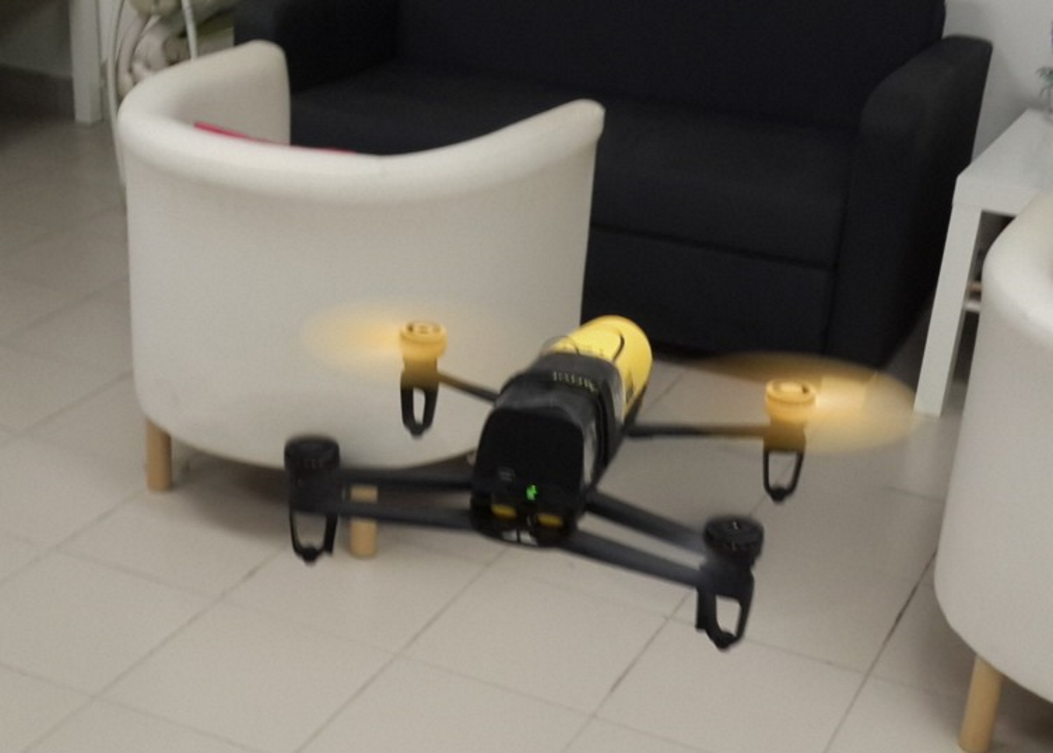
Parrot Bebop drone during flight taken in Advanced Robotic Lab, University of Malaya, Malaysia.

Visual SLAM is the branch where a SLAM system uses a camera as the only extrinsic sensor. It is of special interest as we intend to run the SLAM system on a Micro Aerial Vehicle (MAV) (in Fig 2) due to its agility and freedom of movement in 6-DoF space. However, quad-copters are known to have limited payload capacity and short battery life. Therefore, using a camera is highly practical due to its low power consumption and lightweight. It also provides a rich representation of the environment. Hence, Visual SLAM was chosen to build our system.

**Fig 3:**
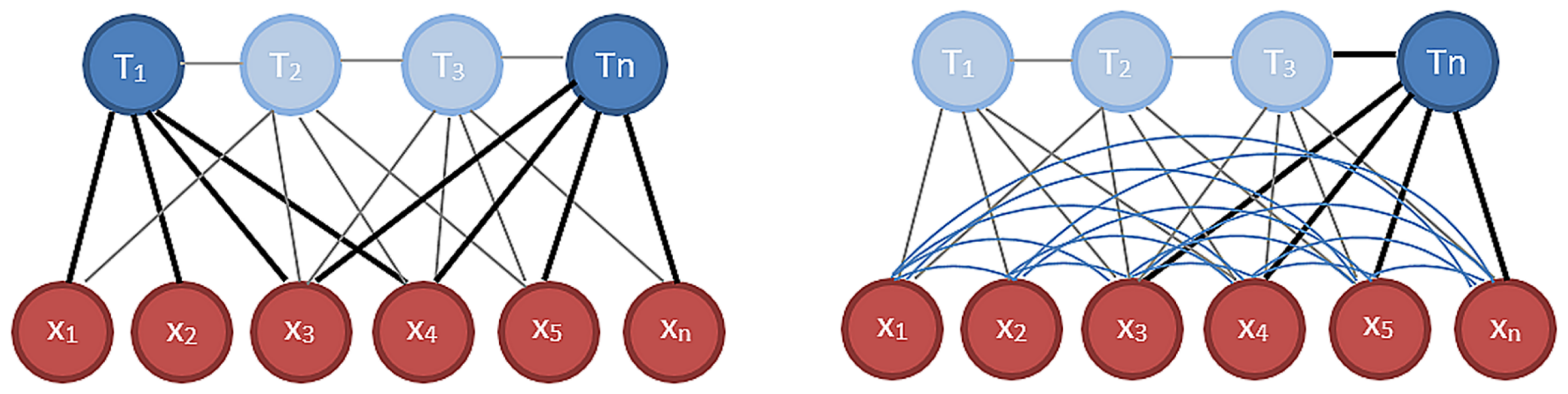
Keyframe BA (left) vs filter based (right): T is a pose in time, x is the feature/landmark—Reproduced from [7].

Many researchers worked on solving SLAM using different sets of robots and sensors. However, there are two dominant approaches to solve it and those are: The Filter-based approach and the Keyframe-based one. (see Fig 3)

- Filtering methods [1–3] summarize the information at each step in a probability distribution. The camera pose **T**_**n**_ is computed using the information of all features of the map.
- Keyframe methods [4–6] retain the optimization approach of global Bundle Adjustment (BA). Instead of relying on all the features in the map, only a few, from certain past frames (aka Key-Frames), are chosen to calculate the current pose **T**_**n**_. This approach is computationally better.

Keyframe based visual SLAM was used in our system based on the work of Strasdat et al. [7]. They performed a thorough analysis of both filter and keyframe based visual SLAM with monocular and stereo rigs in different scenarios and motion patterns, and they found that keyframe BA based SLAM outperformed the filter-based approach in all tests.

In general, all SLAM systems aim to build a globally consistent representation of the environment. The majority, [2, 5, 8–10] including the proposed system, can work on large-scale environments, but they rely on the rigid constraint, while others [11–13] take in consideration the dynamic deformation of shapes to build non-rigid maps, but they can retrieve only a small scale reconstruction. All these systems operate by incorporating tracking, mapping, relocalization after tracking-failure and loop closing. The main block in a SLAM system, specially when operating in a large-scale environment, is loop closure as without loop closures, SLAM will be reduced to odometry [14].

In [15], Castle et. al. used multiple maps concept to extend the work of Klein and Murray on PTAM [4]. However, it does not address the previous principle of loop closing, as the maps, despite being manually initialized, are not merged when they overlap, therefore, the work is reduced to a relocalization rather than building a globally consistent map.

Our approach goes beyond the norm of standard SLAM systems. We argue that relocalization is a *redundant process* that ‘causes’ loss of information. Therefore, we replaced relocalization with Multi-Mapping approach. A new map is generated at the event of tracking loss, this new map is merged with the original map when those two maps intersect using bag of visual words [16].

In this paper, ORB-SLAM [5] was chosen as a base SLAM system due to its robustness and ability to work in real-time indoor or outdoor, as well as its ability to close loops. Hence, we called this implementation of Simultaneous Localization and Multi-Mapping as ORBSLAMM.

Despite the numerous features of ORB-SLAM, the implementation in S1 Link is found to suffer from a number of problems such as the inconsistency in initialization, and the drift caused by pure rotation. This is because ORB-SLAM, in its monocular implementation, needs translation to deduce the depth and scale. In addition, when tracking is lost ORB-SLAM (as other conventional mono-map SLAM systems) attempts to re-localize, by trying to match the current frame with all stored keyframes. Although, it is a very good way to re-localize, it is greedy and, most importantly, it ignores all the information between the location where tracking is lost and the location of re-localization. Furthermore, the strength of loop closing strategies reported in [5], such as Essential Graph optimization, is wasted as it needs an active tracking (frame pose is known) to be performed while in the case of relocalization the PnP (Perspective n Points) method is used to find the pose of the current frame. In this paper the use of multiple maps is introduced by starting a new map at the moment of tracking-loss, and then, merging these maps at the event of loop closure, it is similar to having multiple robots mapping the same environment. Therefore, our proposed system is scalable to work with either one or multiple robots.

**Fig 4:**
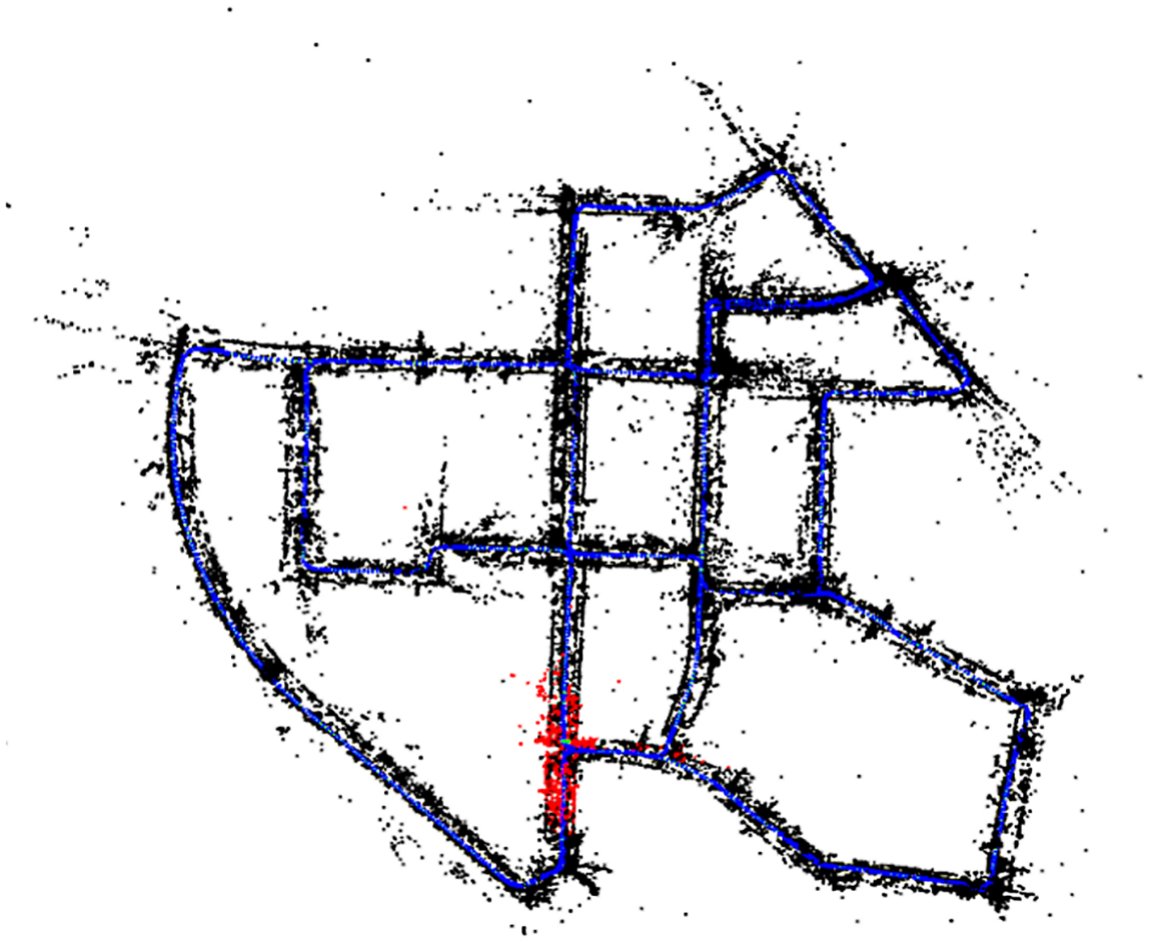
ORBSLAMM running on KITTI sequences 00 and 07 simultaneously. It took 494.2 seconds to get the final map which contains 1934 keyframes, with translation error of 1% of trajectory’s dimensions.

Eade and Drummond [8] worked on the idea of generating a new map at tracking failures and merging them at loop closure events. However, the relations, when closing the loop, were made on node level (keyframe level) rather than propagating the relation to be on cycle level (map level). Also, it is not reported what would happen when the new cycle intersects with older cycles multiple times. This is crucial for optimization. Another problem is that the optimization on cycles is done independently despite the relation between them. This is because of the way the system is built with relations at node level. Moreover, it is not mentioned whether the system is able to merge maps generated by different robots (cameras). The proposed system here is able to propagate the relation to be on map’s level (all its keyframes and map points), and can handle loop closure events happening in the same map and those between different maps. It also enhances map’s accuracy when maps intersect multiple times. Our system is also able to merge maps generated by different cameras thanks to the work in [16]. Fig 4 shows the strength of our proposed system in building a unified global map in real-time of two sequences (00 and 07) of KITTI dataset [17] that were recorded on different days and with different camera calibrations.

Forster et al. [18] worked on the collaborative SLAM problem, where maps generated by multiple robots are merged when they intersect. Their place recognizer accumulates the information with every keyframe added to each map, which reduces the robustness as maps grow. In our system, the new keyframes of each map are compared against *keyframes databases* of other maps. This separation of databases ensures the robustness and real-time operation when searching for potential similarities. The authors in [18] used a down-looking camera that needed a texture-mat to be added in the indoor experiments which limits the application of their system.

Although Howard’s work [3] is done using particle filter with range sensors, the merging methodology of virtual robots traveling “backward in time” is interesting. However, this methodology may cause discrepancy between the virtual robots and actual ones when another overlapping is detected or optimization is performed. Our merging methodology expands on this concept, allowing to update, in real-time, the poses of all keyframes and map points based on the transformation matrix between the matched keyframes of the matched maps. The subsequent keyframes and map points of the matched maps will then be in the common coordinate system.

When the relative poses between different robots/maps is not known a priori, it is necessary to have a loop closure (maps’ intersection) to merge their maps otherwise those maps will remain unconnected. Regardless of this, it is still better in terms of preserving the information after tracking-loss, and these unconnected maps can be utilized in Augmented Reality applications similar to the ones reported in Castle’s work [15].

**Fig 5:**
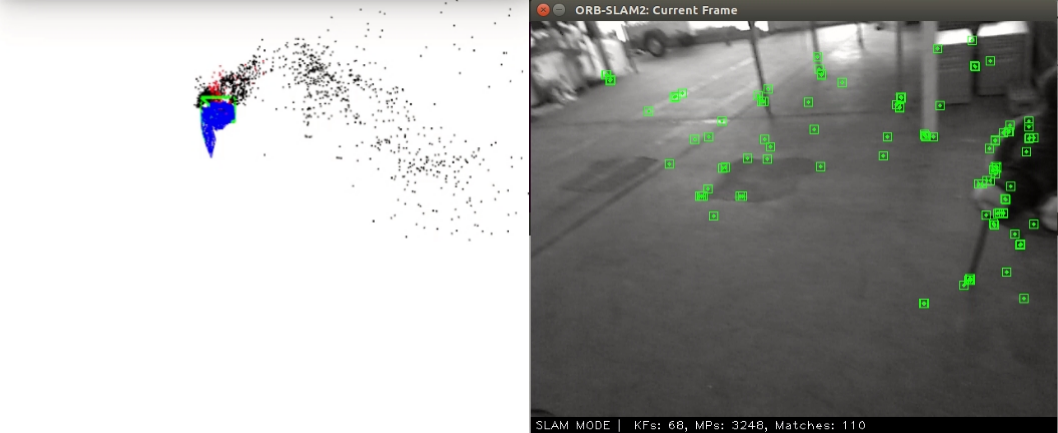
ORB-SLAM getting stuck in wrong initialization *freiburg2_large_with_loop from TUM RGB-D dataset* [19].

Another advantage of the proposed system is the ability to recover from wrong initialization. Fig 5 shows how a wrong initialization may cause the failure of a SLAM system.

In the conventional mono-map SLAM systems after tracking is lost an attempt to relocalize the camera is started, but due to wrong initialization the system gets stuck at the place where it lost tracking, while, in ORBSLAMM a new map is generated enabling tracking and mapping to continue. A comparison video about this issue can be found in S1 Video. Therefore, ORBSLAMM via reinitialization and multi mapping provides the ability to correct errors and recover from failures, and most importantly, preserve the information.

## 2 ORBSLAMM system overview

**Fig 6:**
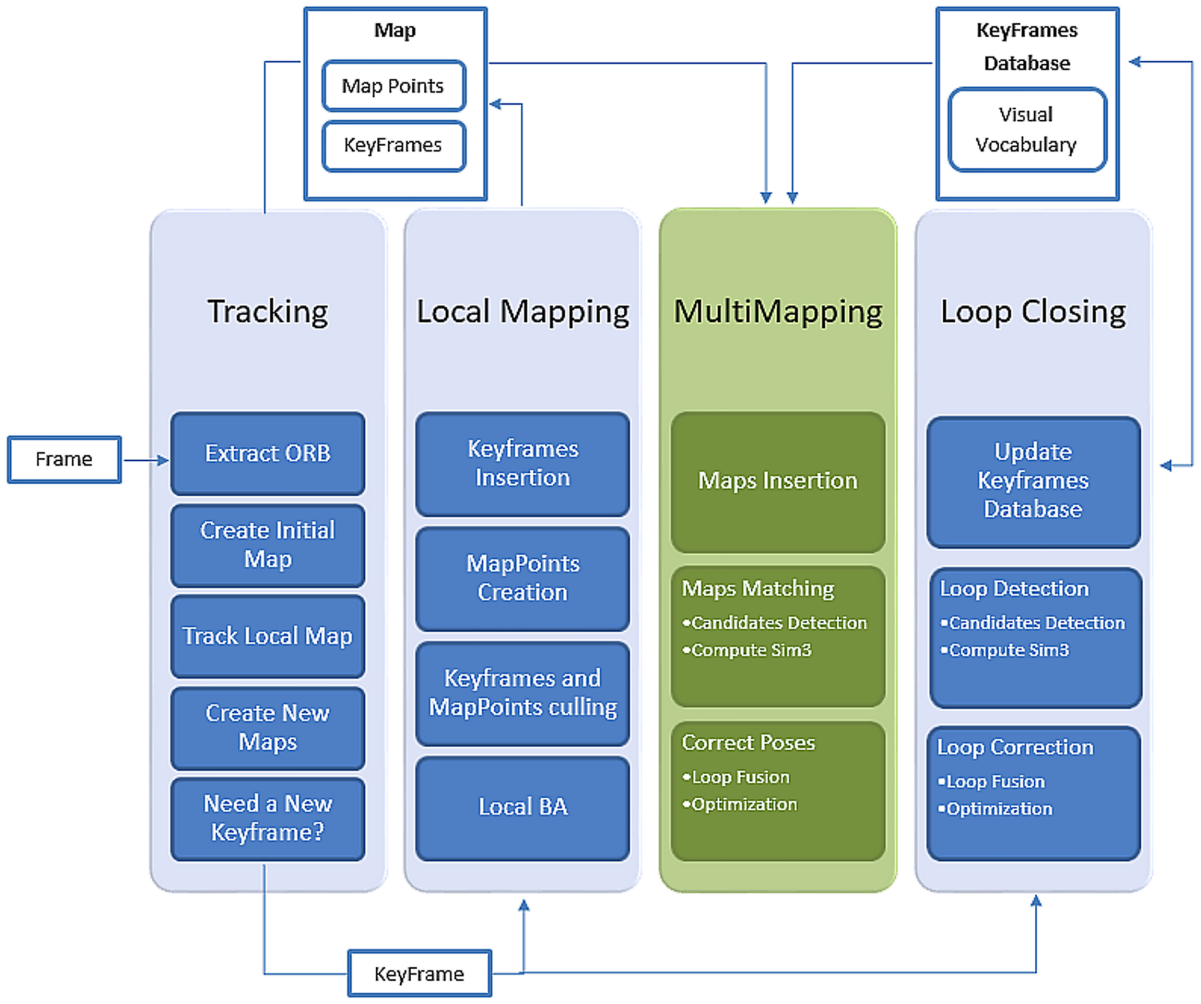
ORBSLAMM system overview.

This section describes the proposed system and its different blocks. It also serves as a road map for this paper. Fig 6 shows an overview of the system, and S1 Fig, in supporting information, provides a better understanding of the different threads in the system and their interactions.

ORBSLAMM is an extension to ORB-SLAM [5]. It preserves the old system which consists of three threads for tracking, mapping and loop closing, and adds a fourth thread that handles the multiple maps generated either by the tracking-thread at the time of tracking-failure, or by running multiple robots to map the environment.

The system starts by grabbing an image frame, then passing it to the tracking thread (presented in section 4) where ORB features are extracted. Once the features are extracted the tracking thread tries to create an initial map by triangulating feature points in the Initializer (aka bootstrapping) (presented in section 3), where it tries to recover the motion and the structure from motion (SfM). ORB-SLAM computes two geometrical models in parallel, the first is a homography assuming a planar scene, and the other is fundamental matrix assuming non-planar scene. After extensive experiments on the public datasets [17, 19, 20] it was found that homography initialization was not contributing in initializing the system, and most of the time the system is initialized correctly using the fundamental matrix. Therefore, homography initialization was removed from the auto Initializer which enhanced the initialization process making it faster and more consistent.

At the time of tracking-failure, the tracking-thread creates a new map and instructs all other threads to *swap* their associations to the newly created map, then it passes the new map to the Multi-Mapping thread (presented in section 7), after that, it starts the automatic bootstrapping. Once the initial map is created and keyframes start to be inserted by local mapping (presented in section 5), the Multi-Mapping thread begins to search for matches between keyframes of the first map and those of the new map using Bag of Words (BoW) [16], similar to the way loop closing thread (presented in section 6) deals with keyframes of the same map. To visualize the new system, maps are processed in a similar manner to keyframes of the conventional mono-map SLAM systems. For more information on tracking, mapping and loop closing refer to [5].

## 3 Initialization

Map initialization is an essential step in monocular SLAM to calculate the depth and retrieve the 3D location of map points and keyframes. It starts by extracting ORB features (**x**_*c*_) in the current frame **F**_*c*_ and tries to match it with the features (**x**_*r*_) in the reference frame **F**_*r*_. The reference frame is initially the first frame. If not enough matches are found, the reference frame is reset. Otherwise, a parallel thread is run to calculate the fundamental matrix **F**_*cr*_:

$$\begin{array}{c} x_{c}^{T}F_{cr}x_{r}=0 \end{array}$$ (1)

Then the Essential Matrix is calculated from the Fundamental Matrix using the Calibration Matrix **K**:

$$\begin{array}{c} E_{rc}=K^{T}F_{rc}K \end{array}$$ (2)

Then the Essential Matrix is decomposed to generate 4 motion hypotheses. These 4 prospective solutions are triangulated and checked to get the best one in terms of the maximum number of points seen in front of both cameras (of **F**_*r*_ and **F**_*c*_) with good parallax and low projection error. If there is no dominant solution with enough triangulated points, the initialization is rejected and the reference frame is reset. Once a solution is chosen, a full BA is performed to refine the initial reconstruction.

## 4 Tracking

The tracking thread is the backbone of the system. It communicates with all other threads and arranges their work. Tracking is started by grabbing an image frame, then extracting FAST corners at eight pyramid levels with a scale factor of 1.2. The number of retained corners is adapted depending on the size of the image and the distribution of the corners inside it. Then, ORB descriptors are computed. After that, the tracking thread checks whether there is an initial estimation of pose, if not, it runs the initializer described in section 3. Once an initial estimation is found, a constant velocity motion model is used to predict the current camera pose.

$$\begin{array}{c} T_{\text{cw}}^{\prime }=M*T_{\text{cw}} \end{array}$$ (3)

where **M** is a 4x4 matrix that encodes the changes in rotation and translation (aka camera motion), it is a member of Lie group **SE(3)**. $T_{\text{cw}}^{{}^{\prime }}$ and **T**_**cw**_ are the poses of the recent frame and the last frame respectively in camera-coordinates *c*.

Map points in the last frame are projected in the current frame and checked for matches. If not enough matches are found (≤ 20) the matcher searches in a wider window. If sufficient matches are found (> 20), the pose of the current frame is optimized and outliers are discarded. Otherwise, an attempt is made to track the pose by matching ORB features of the map points in the current frame with the ones in the reference keyframe. If an initial pose estimation is obtained, the tracker continues by trying to track all points in the local map (refer to the flowchart in S1 Fig). If tracking succeeds, the system checks whether a new keyframe is needed, and instructs the local mapping to process it and add it to the map. Then, the loop closing thread adds the newly processed keyframe to the keyframes database with an inverted index where each visual word in the vocabulary is linked to the list of keyframes it was seen in. This is used to efficiently check for loop closures candidates. However, if tracking fails, a new map is created and passed to the Multi-Mapping thread, and the initialization process in section 3 is rerun.

## 5 Local mapping

The map in ORBSLAMM is a group of keyframes and map points that are located in the world coordinate frame *w*. A map point is the 3D location of an ORB feature that is triangulated and observed by at least three views (keyframes). Map points can be generated by the tracking thread (in section 4) when creating the initial map, and by the local mapping thread. However, the local mapping thread can also remove bad points that have low visibility and are not trackable.

Once the tracking thread finishes the initialization, local mapping starts by inserting the first two views as the initial keyframes. It then continuously checks if the tracking thread has inserted any new keyframes to process them. If there are any, it calculates their BoW structure based on the ORB descriptors generated from the original image frame, then it associates the map points of the original image frame with its keyframe.

The BoW structure contains bags of words vectors, which are samples in the descriptor space (aka visual vocabulary). It helps in map points matching and triangulation. The vocabulary is generated offline using ORB descriptors of features taken from a large set of images. The images should be generic so that the resulted vocabulary can be used with different datasets or environments.

Local Mapping also updates the covisibility graph, which is an undirected weighted graph where each node represents a keyframe and the edges connect keyframes that share a minimum of 15 map points in the local map. The weight of the edge is the number of the shared map points between the two keyframes.

When local mapping is free from processing new keyframes, it performs local BA which optimizes the last processed keyframe and all its neighbors in the covisibility graph.

To summarize, local mapping processes new keyframes and creates new map points. It also removes redundant keyframes and bad map points to maintain a scalable and reliable map. In addition, it updates the covisibility graph among keyframes and runs local BA for optimization.

## 6 Loop closing

This section describes the work of closing loops and correcting the drift in the map. The loop closing thread receives the keyframe generated by the tracking thread after being processed by the local mapping thread, and inserts it into a local database of keyframes. It searches all keyframes in the database that share a word with the current keyframe (the last processed keyframe), using their BoW vectors as described in section 5.

During the loop closing cycle, the loop closing thread checks for new keyframes that are inserted in the local map, then, it queries the keyframes database for a potential matches to the current keyframe. Only those which have a similarity higher than a threshold and are not directly connected to the current keyframe are taken. The threshold is defined by calculating the minimum similarity score between the BoW vector of the current keyframe and all its neighbors in the covisibility graph.

Once a list of candidates is found, a similarity transformation (SIM3) is computed from the current keyframe to each candidate. This gives an insight on the accumulated error. It has the notation 3 as it is calculated in the 3D space *W*, using the 3D to 3D correspondences between the map points seen in the current keyframe and those in the candidate. If these correspondences are more than a threshold (20 matches) a RANSAC solver with 20 iterations is set to find the transformation matrix $T_{c_{2}c_{1}}$. If found, a guided matching and an optimization with all correspondences are performed. If optimization is successful with enough inliers (≥ 20) all the Map points seen in the candidate keyframe and its neighbor keyframes are retrieved and projected using the computed transformation to find more matches:

$$\begin{array}{c} T_{c_{2}w}=T_{c_{2}c_{1}}*T_{c_{1}w} \end{array}$$ (4)

where $T_{c_{2}w}$ is the transformation from world coordinates *w* to candidate’s camera coordinates, $T_{c_{2}c_{1}}$ is the SIM3 transformation from the current keyframe’s camera coordinates to the candidate’s one, and $T_{c_{1}w}$ is the transformation from world coordinates *w* to current keyframe’s camera coordinates. If the total matches were enough (≥ 40), the candidate is accepted as a loop keyframe, and its pose along with the poses of its neighbor keyframes in the covisibility graph and all the map points seen by them are corrected accordingly. Then all map points are fused to remove any redundancy with the other side of the loop, and a new connection in the covisibility graph is established between the two sides of the loop. After that, a graph optimization and global BA using g2o framework [21] is performed to enhance the accuracy of the map.

## 7 Multi-mapping

In this section lies the main contribution of the presented work. It describes the work done in the Multi-Mapping thread and the interactions with other threads to merge maps, and correct *their poses* (i.e. the poses of their keyframes and map points).

### 7.1 Single robot scenario

In a single robot scenario, maps are generated at tracking failure events only, therefore the previous maps are fixed in size (This means that the map which is generated before tracking failure does not grow). The only map that is growing is the current map, where tracking and mapping is running. Each map *M* has an ID, the bigger the ID the newer the map.

When the system starts, it registers the first map *M*_0_ and its Keyframe Database at the multi-mapping thread, at this moment both *M*_0_ and its Keyframe Database are empty. Then, the system fires the tracking-thread (at section 4). As long as tracking is working successfully, the multi-mapper is in an idle state. Once the tracker fails due to a corrupted frame, occlusion or low texture, it creates a new empty map *M*_*n*_, and passes its reference to the multi-mapper along with its Keyframe Database (refer to Fig 6). It also informs local mapper and loop closer to switch their work to the newly created map. After that it tries to initialize the map (as in section 3). Once initialization is successful and new keyframes are started to be processed and registered in the database, the multi-mapper scans for potential matches between these new keyframes of the new map and those stored in the keyframes databases of previous maps. The matching process works in a similar way to the one in section 6.

All the keyframes which are connected to the current keyframe *K*_*c*_ in *M*_*n*_ are retrieved and a minimum similarity score *s*_*min*_ based on their BoW is computed. Then, the multi-mapper loops through the Keyframe Databases of previous maps (*M*_0_ to *M*_*n*−1_) and queries each for matches with the current keyframe *K*_*c*_ imposing the calculated minimum similarity score *s*_*min*_.

For each keyframe *K*_*j*_ of map *M*_*i*_ ∈ [*M*_0_, *M*_*n*−1_] that has more than 15 matching points with *K*_*c*_, a solver to calculate the similarity transformation is set. This transformation contains the information of changes in rotation, translation and scale (7 DoF). Alternatively, RANSAC iterations are performed on each candidate *K*_*j*_, until either a match with enough inliers is found or all candidates fail. If RANSAC returns a similarity transformation, a guided matching is performed to find more correspondences and the transformation is optimized. If enough inliers remain after optimization, *K*_*j*_ is accepted as a loop keyframe.

All the map points which are seen in *K*_*j*_ and its neighbors are retrieved and reprojected on *K*_*c*_ using the computed similarity transformation, then more matches are searched. If the total correspondences were above a threshold (40 matches), the loop is accepted and multi-mapper fuses both sides of the loop and performs a global BA.

#### 7.1.1 Loop fusion

The multi-mapper starts loop fusion by attaching maps (*M*_*n*_ & *M*_*i*_) to each other using the calculated similarity transformation **S**_**cw**_ between *K*_*c*_ and *K*_*j*_. If this was the first fusion between the new map *M*_*n*_ and *M*_*i*_, all the keyframes and map points of *M*_*n*_ are retrieved. Otherwise, only the neighbors of *K*_*c*_ in the covisibility graph are retrieved. Then, the pose of *K*_*c*_ is corrected by setting it to **S**_**cw**_, while the pose of each retrieved keyframe is transformed to *M*_*i*_’s world coordinates using equations in 5

$$\begin{array}{c} T_{ic}=T_{iw}*T_{wc} \end{array}$$ (5a)

$$\begin{array}{c} T_{\text{corr}}=T_{ic}*S_{cw} \end{array}$$ (5b)

Where **T**_**iw**_ is the pose of the retrieved keyframe before correction and **T**_**wc**_ is the inverse pose of *K*_*c*_ before correction. **T**_**corr**_ is the corrected pose of the retrieved keyframe in *M*_*i*_’s world coordinates.

**Fig 7:**
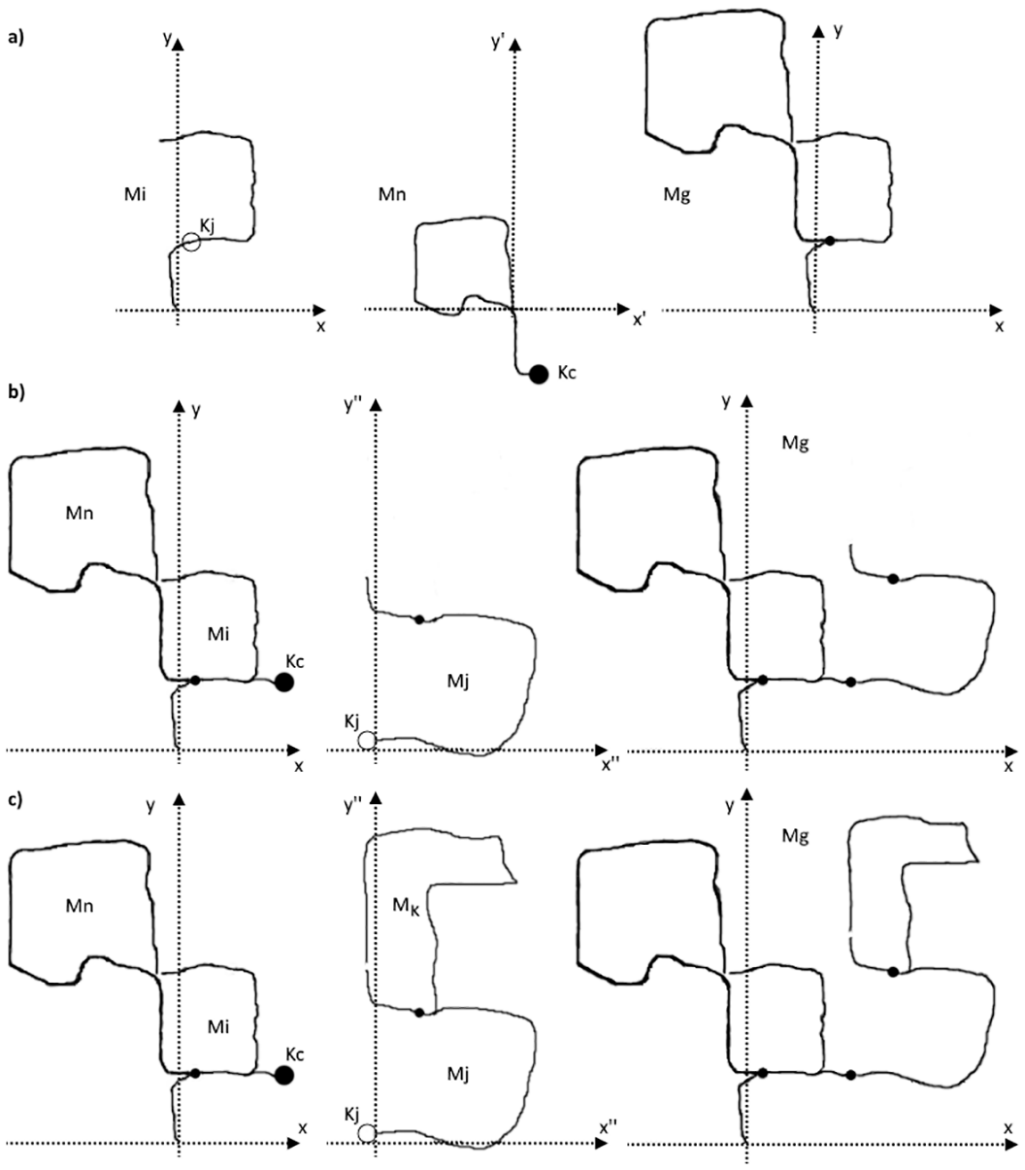
Scenarios of merging the matched maps in the multi-mapper. In the upper row (a) we see the matching between map *M*_*n*_ and map *M*_*i*_ and how the multi-mapper transforms *M*_*n*_ to *M*_*i*_’s coordinates to form a global map *M*_*g*_. In the middle row (b) is the scenario where *M*_*n*_ continues to grow then it intersects with map *M*_*j*_ that is not attached to any other map, so the multi-mapper transforms *M*_*j*_ to *M*_*n*_’s new global coordinates which are map *M*_*i*_’s coordinates. In the lower row (c) is another scenario where map *M*_*n*_ intersects with map *M*_*j*_ which is already matched with another map *M*_*k*_. Here the multi-mapper transforms *M*_*j*_ and all its attached maps (map *M*_*k*_) to *M*_*n*_’s new coordinates. They are now considered the global coordinates. The other option is to transform *M*_*n*_ and *M*_*i*_ to *M*_*j*_’s world coordinates. The solid circles at the end of the line in *M*_*n*_ represent Keyframe *K*_*c*_ while the empty circles in *M*_*i*_ and *M*_*j*_ represent Keyframe *K*_*j*_. The dots show the location where two maps are matched and merged. All transformations are SIM3 (have 7DoF). Notice how the size of the transformed map has changed along with its location and orientation. New maps always start with first Keyframe at (0, 0). These maps are hand drawn and inspired by sequence 00 of the KITTI dataset [17].

All the map points which are observed by each retrieved keyframe and its neighbors are corrected to match the new world-coordinates (refer to Fig 7(a)). Then, the map points observed by *K*_*j*_ and its neighbors are projected into *K*_*c*_ and its neighbors using the corrected poses and the duplicates are fused.

#### 7.1.2 Discussion

If the multi-mapper finds another keyframe *K*_*j*_ of map *M*_*j*_ that matches one of *M*_*n*_ keyframes after being matched with *M*_*i*_ and updated to its world coordinates, then *M*_*j*_’s keyframes and map points are transformed to the ‘*global*’ coordinates of *M*_*i*_ and *M*_*n*_ (Fig 7(b)). If *M*_*j*_ was matched with another map *M*_*k*_, then the ‘*global*’ coordinates could be of either *M*_*j*_ or *M*_*n*_. However, the transformation must be propagated to all attached maps using the respective similarity transformation. Refer to Fig 7(c).

### 7.2 Multiple robots scenario

**Fig 8:**
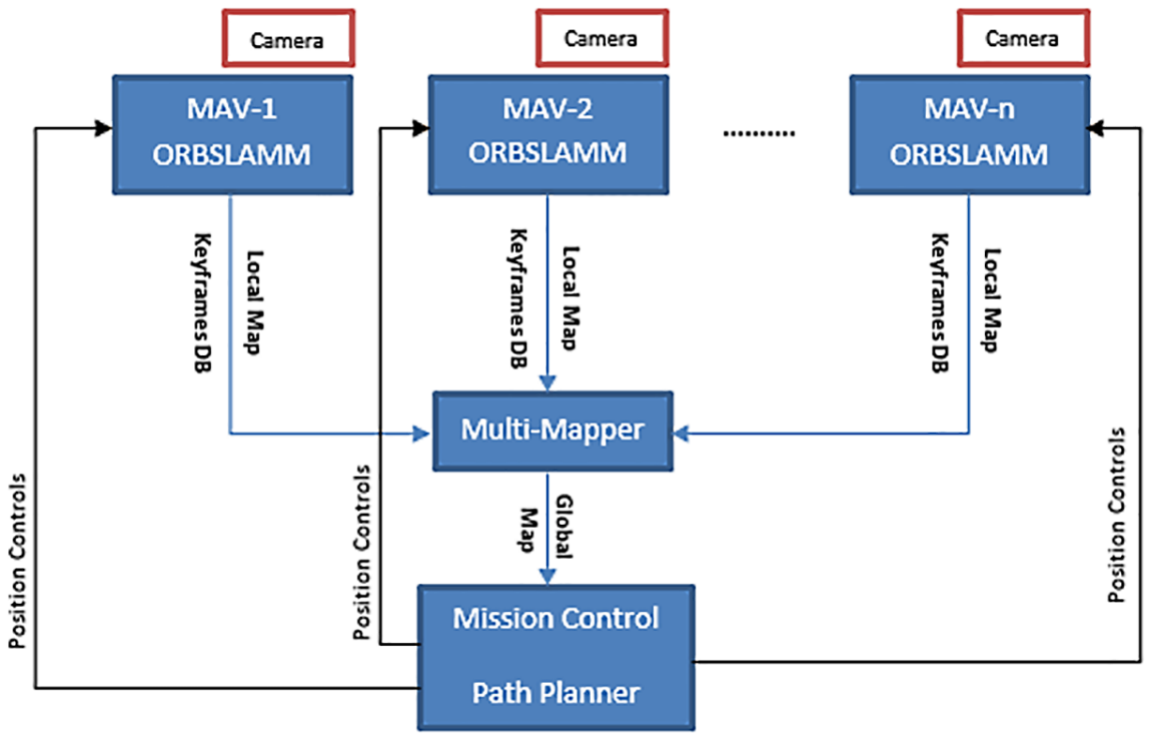
Multiple robots scenario. Each robot has its own ORBSLAMM system running which provides a local map and a keyframe database to the multi-mapper. The multi-mapper tries to merge maps into a global map that can be used by a mission control center to control the position and distribution of the robots.

In a multiple robots scenario, each ORBSLAMM system in each robot starts by registering its initial Map *M*_0_ along with its Keyframe Database at the multi-mapper (Fig 8). The multi-mapper will ensure the assignment of a unique ID to each map. These robots have no prior information of their relative positions in the world. Their ORBSLAMM systems continue the process described in section 7.1, which starts by firing the tracking thread, and then processing new keyframes. Initially the multi-mapper has *n* maps that belong to *n* robots. Unlike the single robot scenario, all maps are growing and being updated simultaneously. Therefore, the multi-mapper continuously checks every map for matches with all other maps. Once a match is found, the multi-mapper transforms the poses of map’s keyframes and map points to the world coordinates of the matched map and attaches both maps to each other. The loop fusion process and the discussion in the single robot scenario (section 7.1) applies in the multiple robots scenario.

If the tracking in any system fails, a new map will be generated and added to the multi-mapper. Therefore, the multi-mapper may have *m* maps that belong to *n* robots, where *m* ≥ *n*.

## 8 Experiments and results

Extensive experiments were performed to test the proposed system. Tests were run on two public datasets in single and multiple robot scenarios. The first dataset is the TUM RGB-D Benchmark [19], which was used to test the speed of initialization and the amount of preserved information. The second is the KITTI dataset [17], which was used to test the amount of preserved information compared to the location where tracking is lost. Evaluation against ground truth is presented using ATE (Absolute Trajectory Error). A comparison between the performance of ORB-SLAM [5], PTAM [4], LSD_SLAM [10], RGBD_SLAM [9] and the proposed system is reported. Some sequences in the KITTI dataset [17], which have loop closures, were modified by adding one blank frame to imitate a corrupted or occluded frame. The system works in real time and at the recorded video frame-rate speed of each dataset. The reported results are the average of several successful tests that were run on a Dell-Precision-M3800 (Intel Core i7-4702HQ CPU @ 2.2GHz x 8 cores with 16 GB RAM) workstation.

### 8.1 TUM’s RGB-D benchmark [19]

This Dataset contains 39 sequences that were recorded in two environments, an office and an industrial hall. All sequences were captured using a hand-held Kinect sensor, except for four sequences, where the Kinect was mounted on a Pioneer 3 robot. The images have a resolution of (640 x 480) and were acquired at 30 fps. The sequences differ in camera motion patterns and speed, the number of loop closures and whether the environment contains static or moving objects. The ground truth trajectories were obtained using eight motion capture cameras with 100 fps.

**Fig 9:**
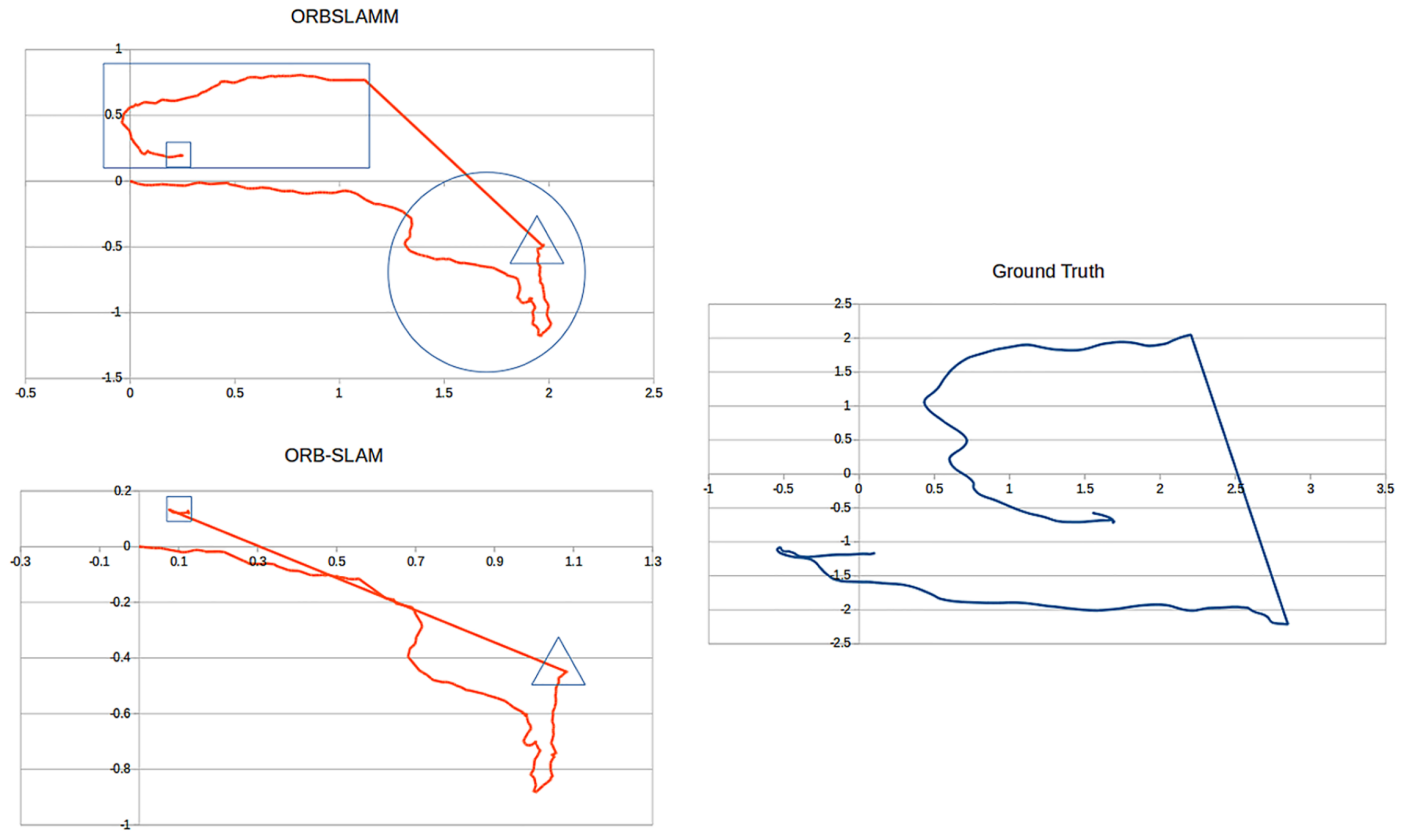
Comparison between ORBSLAMM and ORB-SLAM on the sequence *freiburg2_large_with_loop* without alignment or scale correction. The portion of trajectory shown in rectangle (Map *M*_1_) is completely missed in ORB-SLAM because of the relocalization approach. The portion in the circle is missing in the ground-truth due to a limited number of motion capture cameras. The straight line is where tracking is lost due to a low number of features (wall). The triangles mark the beginning of the wall and the tracking-loss. The square in ORB-SLAM marks the relocalization, and the one in ORBSLAMM marks the loop closure and similarity transformation from *M*_1_ to *M*_0_.

The most interesting sequence in this dataset is *freiburg2_large_with_loop* (Fig 9) because in the middle of the trajectory a wall (no texture) is observed which causes the loss of tracking. After passing this wall the environment returns to have enough features for a successful tracking. Later the camera closes the loop by returning to the starting point. However, the conventional mono-map SLAM systems, as in ORB-SLAM, fail to map the portion between tracking loss and loop closing, despite its richness in trackable features. This sequence was one of the main motivations to work on ORBSLAMM. In Fig 9, the portion in the rectangle is the second map *M*_1_ which is successfully transformed to *M*_0_ coordinates after the loop closure.

**Fig 10:**
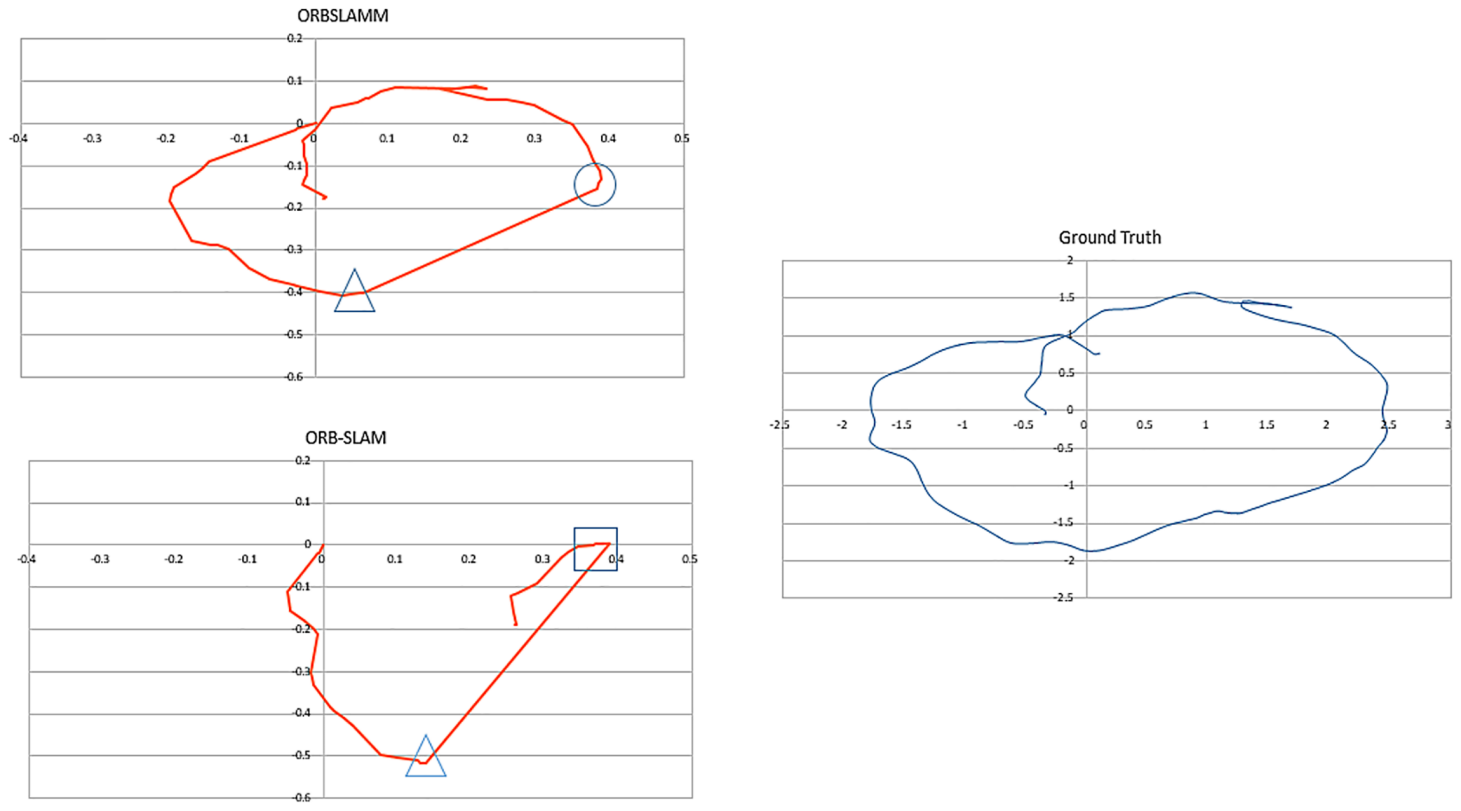
Comparison between ORBSLAMM and ORB-SLAM on the *freiburg2_360_kidnap* sequence without alignment or scale correction. The triangle marks the moment of the kidnap. The circle marks the first keyframe in the second map *M*_1_ of ORBSLAMM, transformed to map *M*_0_’s world coordinates after the loop closure. The square marks the relocalization keyframe of ORB-SLAM. Ground truth trajectory is shown for reference on the accuracy and data preservation of ORBSLAMM.

In a similar case, the sequence of *freiburg2_360_kidnap* (Fig 10) where the camera is moved in a circular trajectory and in the middle of its movement the camera is covered for some time (kidnapped). This causes the tracking loss and, subsequently, the loss of all information until the camera returns to its starting point (closes the loop) where the relocalization retrieves the camera pose to continue mapping and tracking. However, and as reported in [5], ORB-SLAM has worse RMSE than PTAM [4] and that is because relocalization does not correct the drift. On the other hand, both fail to retrieve the information between tracking-loss and loop closure. Figs 9 and 10 show the superior results of ORBSLAMM over ORB-SLAM in terms of information preservation. In Fig 10, the difference in the beginning of the trajectory (Trajectories start at 0) is because ORBSLAMM initializes faster and therefore it registers more information, and also because it performs bundle adjustment after loop closure. S2 Video shows the difference in action. S2 Fig shows the comparison against ground-truth after alignment and scale correction.

**Fig 11:**
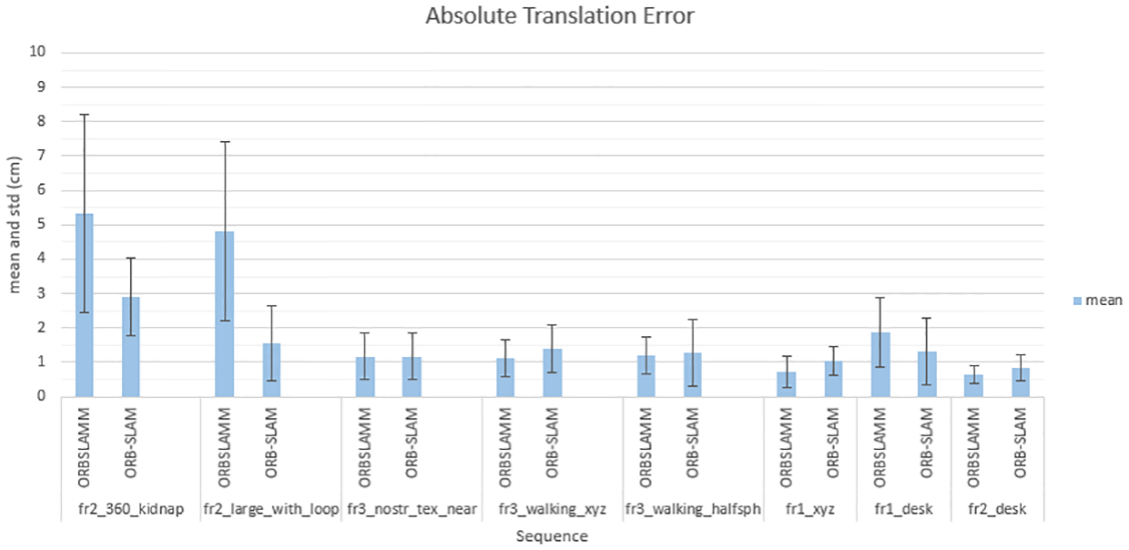
Comparison of absolute translation error’s mean and standard deviation. Comparing the mean and standard deviation of the absolute translation error between our approach and ORB-SLAM using TUM-RGBD benchmark [19]. *fr1_floor* and *fr3_nostr_tex_far* sequences are not reported because ORB-SLAM fails to initialize. RMSE and other comparison information are reported in Table 1.

Table 1 contains the mean results of 5 successful experiments due to the fact that ORB-SLAM has a frequent inconsistency in initialization. For instance, in the experiment *fr3_walking_xyz*, it took 10 runs to get 5 initializations and one of them has a wrong planar with a mean-time to initialize equals to 15.85 seconds. While in ORBSLAMM, using the fundamental matrix only, the first 5 runs were successful with correct initialization and the mean-time to initialize is 5.77 seconds. Note that, generally, the number of keyframes can not be used to measure the amount of information as ORB-SLAM (and ORBSLAMM) has a culling algorithm that removes redundant Keyframes which do not hold new information (as in sequences *fr2_desk* and *fr3_walking_halfsphere*). However, since it is the same algorithm in both systems, the additional keyframes in ORBSLAMM (as in sequences *fr2_360_kidnap* and *fr2_large_with_loop*) are coming from the faster initialization and the data registered after tracking loss, therefore, they hold new information as clearly visible in Figs 9 and 10. The difference between number of keyframes and the matched ones in *fr2_large_with_loop* is because the ground-truth information is available only for the beginning and end of the trajectory. The trade-off is calculated by dividing the error by the number of keyframes. It shows that ORBSLAMM preserves more information without affecting the accuracy. Fig 11 complements Table 1 by comparing the mean and standard deviation of our approach against ORB-SLAM in the sequences that both run successfully.

**Table 1:** Comparison between ORBSLAMM and ORB-SLAM in a single robot scenario in TUM RGB-D benchmark [19].

	ORBSLAMM				ORB-SLAM			
Sequence	Trade-off	RMSE	nKFs	TTI	Trade-off	RMSE	nKFs	TTI
fr2_360_kidnap	0.09	6.06	**64(64)**	**1.67**	0.09	**3.12**	33(33)	2.38
fr2_large_with_loop	0.07	5.4	**272(75)**	**2.57**	**0.05**	**1.9**	210(34)	11.55
fr3_nostr_tex_far	**0.06**	**2.12**	**33(32)**	**2.5**	x	x	x	x
fr3_nostr_tex_near	0.02	1.35	62(61)	3.09	0.02	1.35	62(61)	**3.07**
fr3_walking_xyz	**0.03**	**1.20**	**32 (32)**	**5.77**	0.04	1.4	30 (30)	15.13
fr3_walking_halfsph	0.05	2.24	43(43)	**9.66**	**0.038**	**1.7**	**44(44)**	13.39
fr1_xyz	**0.02**	**0.85**	**31(31)**	**0.41**	0.04	1.12	31(29)	7.53
fr1_floor	**0.01**	**1.81**	**133(128)**	**1.04**	x	x	x	x
fr1_desk	0.02	2.11	**80(80)**	**1.5**	0.02	**1.62**	67(67)	4.5
fr2_desk	0.00	**0.69**	163(124)	**2.10**	0.00	0.92	**164(125)**	5.6

RMSE is the ATE (Absolute Trajectory Error in cm), nKFs is the number of keyframes and TTI is the Time To Initialize (in seconds), Trade-off is $\frac{RMSE}{nKFs}$. The number of keyframes between brackets are the ones matched with the ground truth using the ‘evaluate_ate_scale’ tool which is an upgrade of the ‘evaluate_ate’ tool provided by the benchmark. It aligns trajectory with ground-truth using a similarity transformation based on the method of Horn [22]

We also ran the comparison tests on sequences *sit_halfsph, sit_xyz, str_tex_near, str_tex_far* but the results were similar in both systems. However, in *sit_halfsph* ORB-SLAM tends to initialize a corrupted planar, while ORBSLAMM always initializes correctly.

In addition to ORB-SLAM [5], we used this Benchmark to compare our work with other state-of-the-art systems, namely, PTAM [4], LSD_SLAM [10] and RGBD_SLAM [9]. In Table 2 we report the RMSE results of the absolute translation error of these tests. We built on the information reported in Table 3 (of [5], page 11). We added an extra sequence (*fr2_large_with_loop*). We also added the results of RGBD_SLAM on the fr3 dataset and updated the results of some sequences where our tests showed different values than the ones reported in the aforementioned table. We ran the tests using the source code of PTAM and ORB-SLAM, and the ROS implementation of LSD_SLAM and RGBD_SLAM. We found that LSD_SLAM performs poorly in initializing the system and it, along with PTAM, fail to relocalize in both *fr2_360_kidnap* and *fr2_large_with_loop* sequences. On the other hand, RGBD_SLAM performed well on all sequences, retrieving a rich representation of the environment, however, its accuracy was lower than the one of the sparse based approaches and ran much slower. Although RGBD_SLAM uses the depth information to retrieve the true scale, however, we also reported its results after performing 7DoF alignment with ground-truth in a similar way to the results reported by other solutions.

**Table 2:** Comparison of RMSE of the absolute translation error in a single robot scenario among state-of-the-art systems using TUM RGB-D benchmark [19].

	ORBSLAMM	ORB-SLAM	PTAM	LSD_SLAM	RGBD_SLAM
fr2_360_kidnap	6.06	3.12	**2.63**	X	47.95(44.95)
fr2_large_with_loop	5.4	**1.9**	X	X	42.12(32.02)
fr3_nostr_tex_far	**2.12**	X	4.92	18.31	7.03(5.50)
fr3_nostr_tex_near	1.35	1.35	2.74	7.54	4.73(2.26)
fr3_walking_xyz	**1.2**	1.4	X	12.44	2.97(2.77)
fr3_walking_halfsph	2.24	**1.7**	X	X	4.82(4.82)
fr1_xyz	**0.85**	1.12	1.15	9.00	1.34(1.34)
fr1_floor	**1.81**	X	X	38.07	3.51(3.51)
fr1_desk	2.11	**1.62**	X	10.65	2.58(2.52)
fr2_desk	**0.69**	0.92	X	4.57	9.5(3.94)

RMSE values are in (cm). The values of ORBSLAMM, ORB-SLAM, PTAM and LSD_SLAM are after performing 7DoF alignment with the ground-truth. X means the system failed in initialization or lost tracking early in the sequence. The values of RGBD_SLAM is after applying 6DoF alignment with ground-truth while the numbers between brackets are after applying 7DoF alignment. The larger difference in RMSE between our approach and ORB-SLAM in sequences *fr2_Large_with_loop* and *fr2_360_kidnap* is due to the extra information retrieved by our system (Refer to Table 1).

#### 8.1.1 Multi-robots

In the multi-robots scenario, we used two threads to imitate two robots. We provided each thread with half of the sequence, then we created the multi-mapper thread and linked both robots (threads) to it. After that, we triggered all seven threads (3 for Robot-1, 3 for Robot-2 and the multi-mapper). We ran the experiments on three sequences of the dataset that contain a loop closure. Table 3 shows the comparison results between ORBSLAMM and ORB-SLAM. ORBSLAMM with two robots takes half the time taken by ORB-SLAM to finish each sequence while retrieving double the amount of information with a notably better trade-off in terms of accuracy relative to preserved information.

**Table 3:** Comparison between ORB-SLAM and ORBSLAMM in a multi-robot scenario.

	ORBSLAMM				ORB-SLAM			
Sequence	Trade-off	RMSE	nKFs	TTF	Trade-off	RMSE	nKFs	TTF
fr2_large_with_loop	**0.088**	6.76	**315(76)**	**125.28**	0.192	**6.56**	210(34)	216.11
fr2_360_kidnap	**0.070**	4.42	**63(63)**	**30.92**	0.097	**3.21**	33(33)	69.27
fr3_nostr_tex_near	0.026	1.66	62(62)	**37.04**	0.026	**1.60**	62(61)	73.56

RMSE is the ATE (in cm), nKFS is the number of keyframes and TTF is the Time To Finish the sequence (in seconds) excluding loading time of ORB vocabulary, Trade-off is $\frac{RMSE}{nKFs}$, in TUM RGB-D benchmark [19]. The number of keyframes between brackets is the one matched with the ground truth using the evaluate_ate tool provided by the benchmark

**Fig 12:**
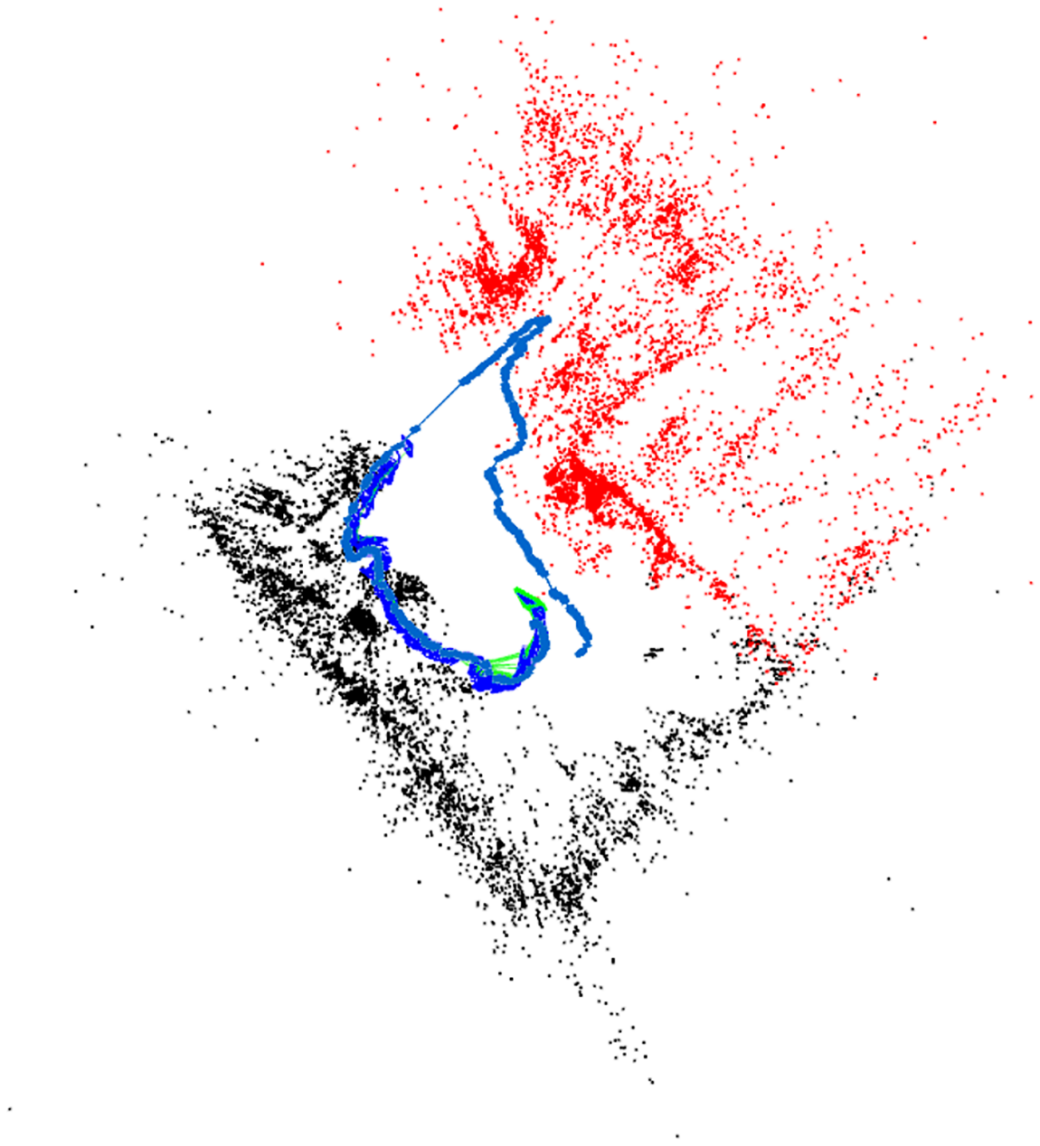
ORBSLAMM in multi-robot scenario while running on *fr2_large_with_loop* sequence. Two robots (threads) were run simultaneously with no prior knowledge of their relative poses. The thin-blue is the trajectory of Robot-1 (*M*_0_) and the thick-blue is the trajectory of Robot-2 (*M*_1_) after closing the loop and performing similarity transformation to *M*_0_ world-coordinates. The green square is the current keyframe *K*_*c*_ in *M*_1_. Note that all the features from *M*_0_ are now *visible* to *K*_*c*_ (in red color). The line between *M*_0_ and *M*_1_ with no features is where the wall is located. The green lines link keyframes in the co-visibility graph.

Fig 12 shows the result of running two threads on *fr2_large_with_loop* sequence, these two ‘robots’ were run simultaneously with no prior knowledge of their relative initial poses. Each one was given half of the sequence’s images. The mean tracking time is 38 milliseconds per frame, and total time to finish the sequence is 2 minutes and 44 seconds (164.075 seconds) including the time required to load the ORB vocabulary and 125.289 seconds excluding vocabulary loading time, compared to ORB-SLAM’s 225.916 seconds and 216.113 seconds respectively. This map contains 365 keyframes compared to 210 keyframes in ORB-SLAM due to its inability to map the thick-blue portion with its relocalization approach.

### 8.2 KIT’s KITTI dataset [17]

The KITTI odometry dataset contains 22 sequences, 11 of them are available with ground truth for training/tuning and the other 11 are used for evaluation. These sequences were recorded using a car equipped with multiple sensors. The car was driven in different environments like inside a city, on the highway and in rural areas. Besides environment, these sequences differ in the length of the trajectory, the speed of driving, the number of static and moving objects (e.g. cars, pedestrians) and the number of loop closures. The images were recorded at 10 fps with a resolution of (1226 x 370). The ground-truth trajectories were obtained using a Velodyne laser scanner and a GPS.

ORB-SLAM performs well in the KITTI Dataset, and specifically in the sequences that have loop closures. Aside from the sequences 01 and 08, the trajectory error in sequences (00 to 10) is around 1% of the trajectory’s dimensions. Therefore, in each sequence that has a loop closure we added a single blank frame to imitate occlusion or camera malfunction. Due to fast movement of the car and low frame rate of 10 fps, one blank-frame was sufficient to cause tracking-loss. To test the accuracy and data preservation of our system against conventional mono-map SLAM systems we changed the position of this blank frame to be at (10%, 50% and 90%) of the trajectory’s total frames number. Table 4 shows the modified sequences and the frame that was replaced by the blank-frame in each sequence and at each percentage. Table 5 shows the results in comparison with the performance of ORB-SLAM. S3–S8 Figs, in supporting information, show the results graphically.

**Table 4:** The modified KITTI dataset.

Sequence	Frames #	10%	50%	90%
00	4541	000454	002270	004086
02	4661	000466	002330	004215
05	2761	000276	001380	002485
06	1101	000110	000550	000991
07	1101	000110	000550	000991
09	1591	000159	000795	001432

Frames# is the total number of frames in the sequence. 10%, 50% and 90%, show the frame that was replaced by the blank-frame to induce tracking loss.

**Table 5:** Comparison between ORB-SLAM and ORBSLAMM Results on the different sequences of the Modified KITTI dataset.

	ORBSLAMM						ORB-SLAM					
Seq.	10%		50%		90%		10%		50%		90%	
	nKFs	RMSE	nKFs	RMSE	nKFs	RMSE	nKFs	RMSE	nKFs	RMSE	nKFs	RMSE
00	**1618**	**4.93**	**1641**	7.41	**1615**	9.14	1346	5.84	1574	**5.38**	1529	**7.61**
02	**1964**	19.48	**2251**	**23.43**	**2227**	**35.8**	245	**1.11**	1306	28.37	1867	36.68
05	**1007**	5.33	**979**	**4.3**	**1015**	**5.54**	231	**4.01**	958	4.66	973	5.59
06	**427**	13.59	**399**	35.42	**425**	**12.19**	147	**0.35**	273	**30.14**	420	17.73
07	**400**	12.22	**414**	19.49	**420**	**1.72**	51	**0.27**	218	**8.41**	377	18.79
09	**688**	37.95	**752**	36.74	**743**	53.67	82	**0.63**	381	**3.78**	675	**44.77**

RMSE is the Absolute Trajectory Error in meters (m), nKFs is the number of keyframes. The 10%, 50% and 90% are the locations of the corrupted frame (blank-frame) relative to sequence’s total number of frames (refer to Table 4).

**Fig 13:**
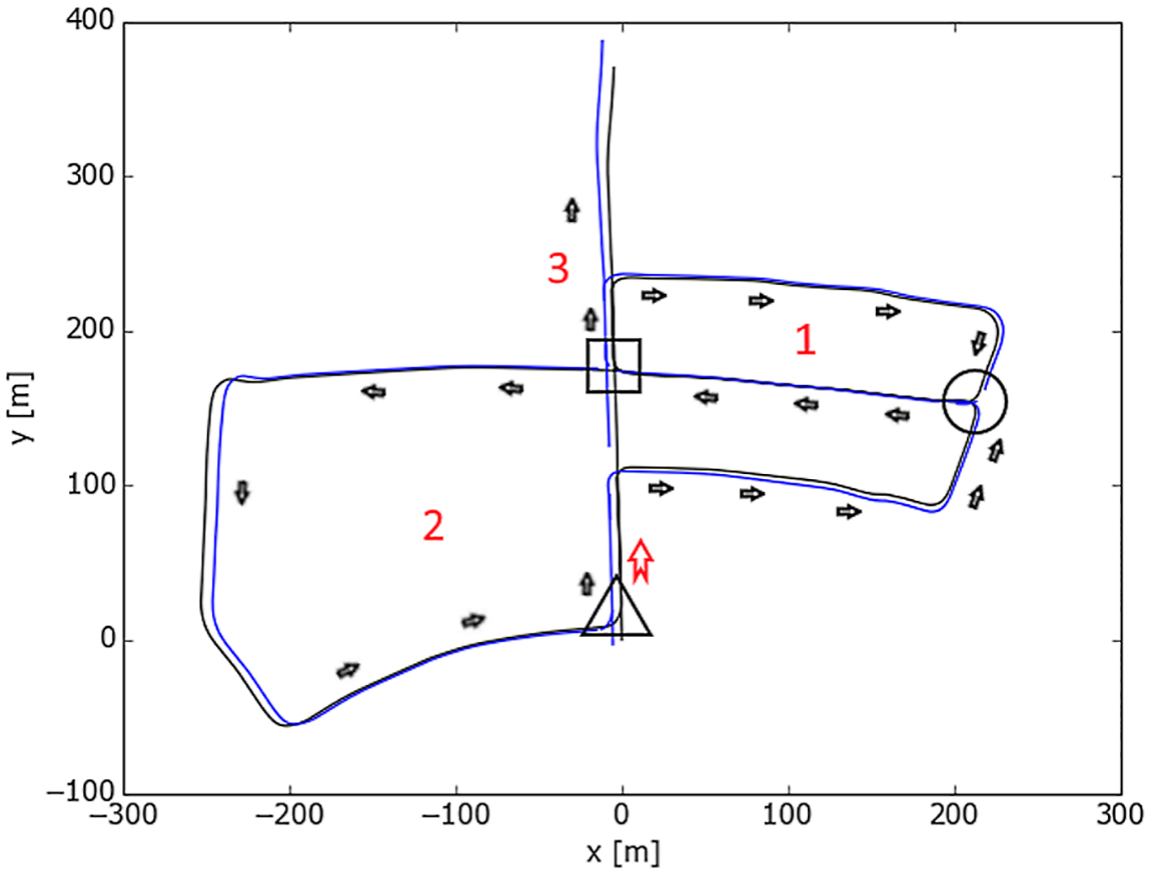
KITTI sequence 05 loops. The circle marks the first loop closure. The triangle marks the second and the square marks the third loop closure. The unique red arrow marks the beginning of the sequence. The black arrows show the direction of movement.

In sequence 05, the corrupted frames at 50% and 90% (001380 and 002485 respectively) come right after the first and second loop closures (Fig 13 shows the locations of the loops), therefore ORB-SLAM is able to relocalize immediately. The lower number of keyframes is due to the culling algorithm, so no information is missing. If we change the location of the corrupted frame to be before the second loop closure (frame 002420) the nKFs in ORB-SLAM, slightly, drops to 971 but the RMSE rises to 15.55 m while in ORBSLAMM the nKFs remains high at 1017 and the error rises to 13.37 m.

#### 8.2.1 Multi-robots

**Fig 14:**
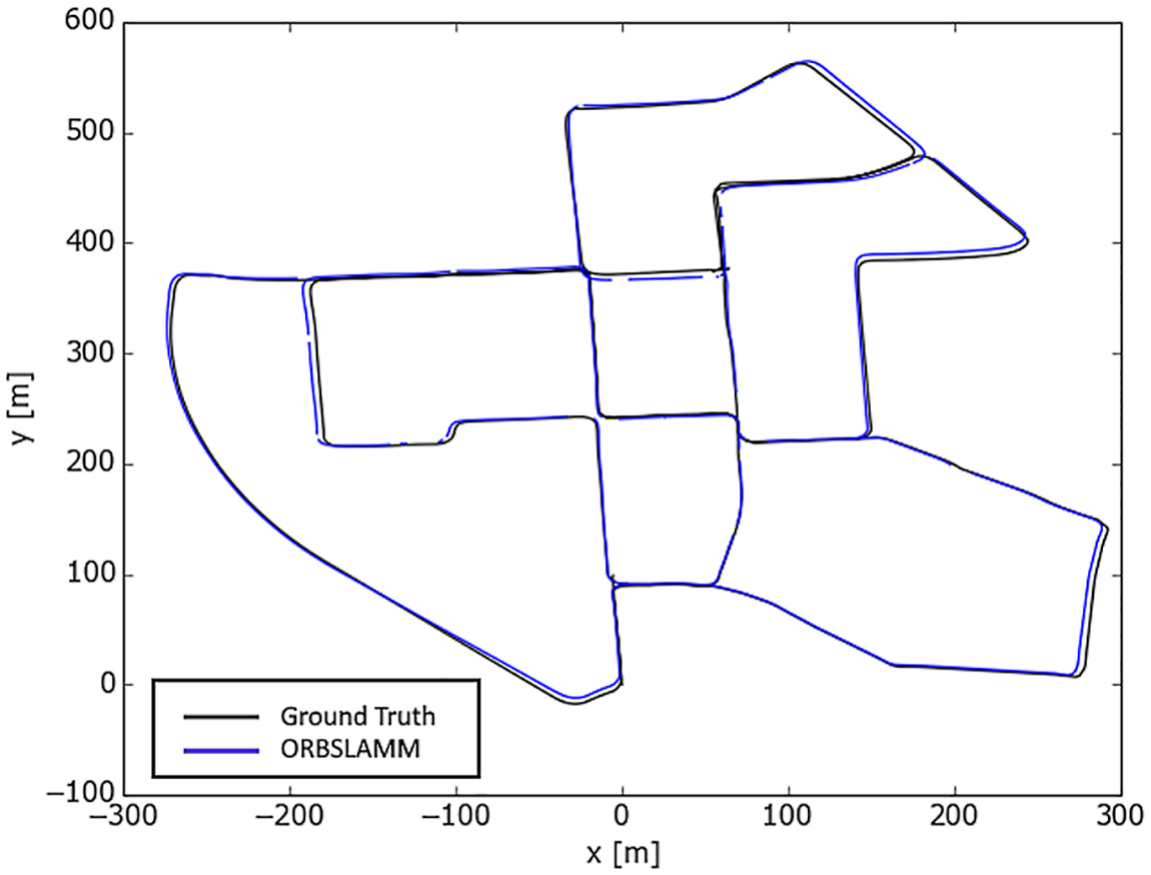
ORBSLAMM running on KITTI sequences 00 and 07 simultaneously. The ground truth of sequence 07 was translated to the correct location relative to sequence 00. ORBSLAMM successfully merged both sequences in one map and in real-time. It took 494.2 seconds to get the final map which contains 1934 keyframes, with translation error of 1% of trajectory’s dimensions.

We ran two threads, each mapping a different sequence (00 and 07) simultaneously. Even though the sequence-07 was recorded on 30th of September 2011 and the sequence-00 was recorded 3 days later, on 3rd of October 2011, and despite the fact that each sequence has different calibration settings, the multi-mapper detected the overlapping portion and merged both sequences in one map. The total time to get the full map of **1934** keyframes is **494.2** seconds, with RMSE of **7.13(m)** which is around **1%** of trajectory’s dimensions (564m x 580m). Fig 14 shows the resulted trajectory against the ground-truth of both sequences. The ground truth of Sequence 07 was translated to its correct location relative to sequence 00. The timestamps of sequence 07’s ground truth and Map1’s keyframes were made continuous to the ones in sequence 00 to enable running the comparison program.

## 9 Conclusion and discussion

We have presented the concept of Simultaneous Localization and Multi-Mapping (SLAMM) where a new map is generated when tracking fails, later these maps are merged when they intersect in a loop closure event. We showed the results of the proposed system on public datasets in indoor and outdoor sequences with hand-held and car-mounted cameras. We also showed the ability of our system to map and merge multiple large scale sequences (00 and 07) from the KITTI dataset [17] that share a portion of the trajectory, in real-time, despite the difference in recording days and camera calibrations. This was possible, thanks to the excellent work of Mur-Artal et al. on ORB-SLAM [5] which we used as a base SLAM system and hence we named this solution as ORBSLAMM. We also presented the comparison results between the proposed system and ORB-SLAM, in terms of accuracy and information preservation, on a modified version of the KITTI dataset where a blank-frame was added at different locations (10%, 50% and 90% of the trajectory’s size) to imitate occlusion or sensor malfunction. We also presented the flexibility of our system to handle maps generated by single robot due to tracking failures or by multiple robots by dividing the sequences among multiple threads which reduces the time required to finish the sequence without compromising the accuracy.

In this work, we argued that relocalization approach *causes* information loss and reduces map’s accuracy, and we proved this argument using the modified KITTI dataset and TUM RGB-D benchmark [19] specifically in the sequences *fr2_large_with_loop* and *fr2_360_kidnap*. We also showed that using only the fundamental matrix to initialize the map is sufficient and enhances the speed and consistency of operation. One case that relocalization can outperform the proposed system is when the robot is kidnapped to a previously visited location as the PnP (Perspective n Points) approach will be able to retrieve the pose faster than our solution that needs to initialize the map first (perform bootstrapping). Therefore, one may argue in favor of keeping the relocalization running until a new map is initialized.

The reinitialization and multi-mapping approach is able to recover from failures whether in map’s initialization or tracking; leading to information preservation by maximizing the reconstructed portion of the environment.

### 9.1 Future work

In the proposed system we use the camera as the only sensor to observe and reconstruct the environment, therefore, maps’ intersection is necessary to merge them as we do not know their relative positions a priori. If maps do not intersect they will remain disconnected, this limitation in a single robot scenario (maps generated at tracking failure events) can be solved by exploiting the Inertial Measurement Unit (IMU) of the robot. Therefore, a work on iORBSLAMM (inertial ORBSLAMM) is ongoing and will enable connecting the multiple maps generated at tracking-loss events without the need for loop closures (Maps’ intersections).

## Supporting information

S1 Link https://github.com/raulmur/ORB_SLAM2. (TXT)

S1 Video ORBSLAMM vs. ORB-SLAM in fr2_large_with_loop with wrong initialization. Please note that the corrupted planar initialization is caused by using the dual model initialization of ORB-SLAM (i.e Homography and Fundamental Matrix models). ORBSLAMM uses only the fundamental matrix model and thus, does not suffer from this error. However, we enabled the Homography model to initiate this error and demonstrate the robustness of our approach, in terms of recovery from failure, in comparison to the state-of-the-art.
(MP4)

S2 Video ORBSLAMM vs. ORB-SLAM in fr2_360_kidnap. (MP4)

S1 Fig ORBSLAMM system flowchart. (TIF)

S2 Fig ORBSLAMM vs. ORB-SLAM in TUM RGB-D. Comparison against ground-truth after alignment and scale correction. The upper row is sequence *fr2_360_kidnap* and the lower row is sequence *fr2_large_with_loop*. The left column is for ORBSLAMM while the right column is for ORB-SLAM.
(TIF)

S3 Fig ORBSLAMM vs. ORB-SLAM in the modified KITTI dataset—Sequence 00. The first row is for error at 10%, the second row is for error at 50% and the third row is for error at 90%. The left column is for ORBSLAMM and the right column is for ORB-SLAM.
(TIF)

S4 Fig ORBSLAMM vs. ORB-SLAM in the modified KITTI dataset—Sequence 02. The first row is for error at 10%, the second row is for error at 50% and the third row is for error at 90%. The left column is for ORBSLAMM and the right column is for ORB-SLAM.
(TIF)

S5 Fig ORBSLAMM vs. ORB-SLAM in the modified KITTI dataset—Sequence 05. The first row is for error at 10%, the second row is for error at 50% and the third row is for error at 90%. The left column is for ORBSLAMM and the right column is for ORB-SLAM.
(TIF)

S6 Fig ORBSLAMM vs. ORB-SLAM in the modified KITTI dataset—Sequence 06. The first row is for error at 10%, the second row is for error at 50% and the third row is for error at 90%. The left column is for ORBSLAMM and the right column is for ORB-SLAM.
(TIF)

S7 Fig ORBSLAMM vs. ORB-SLAM in the modified KITTI dataset—Sequence 07. The first row is for error at 10%, the second row is for error at 50% and the third row is for error at 90%. The left column is for ORBSLAMM and the right column is for ORB-SLAM.
(TIF)

S8 Fig ORBSLAMM vs. ORB-SLAM in the modified KITTI dataset—Sequence 09. The first row is for error at 10%, the second row is for error at 50% and the third row is for error at 90%. The left column is for ORBSLAMM and the right column is for ORB-SLAM.
(TIF)
