# Supplementary figures and images for: SLAMM: Visual monocular SLAM with continuous mapping using multiple maps
Source: PLoS One. 2018 Apr 27;13(4):e0195878. doi: 10.1371/journal.pone.0195878 (PMC5922523; doi:10.1371/journal.pone.0195878)

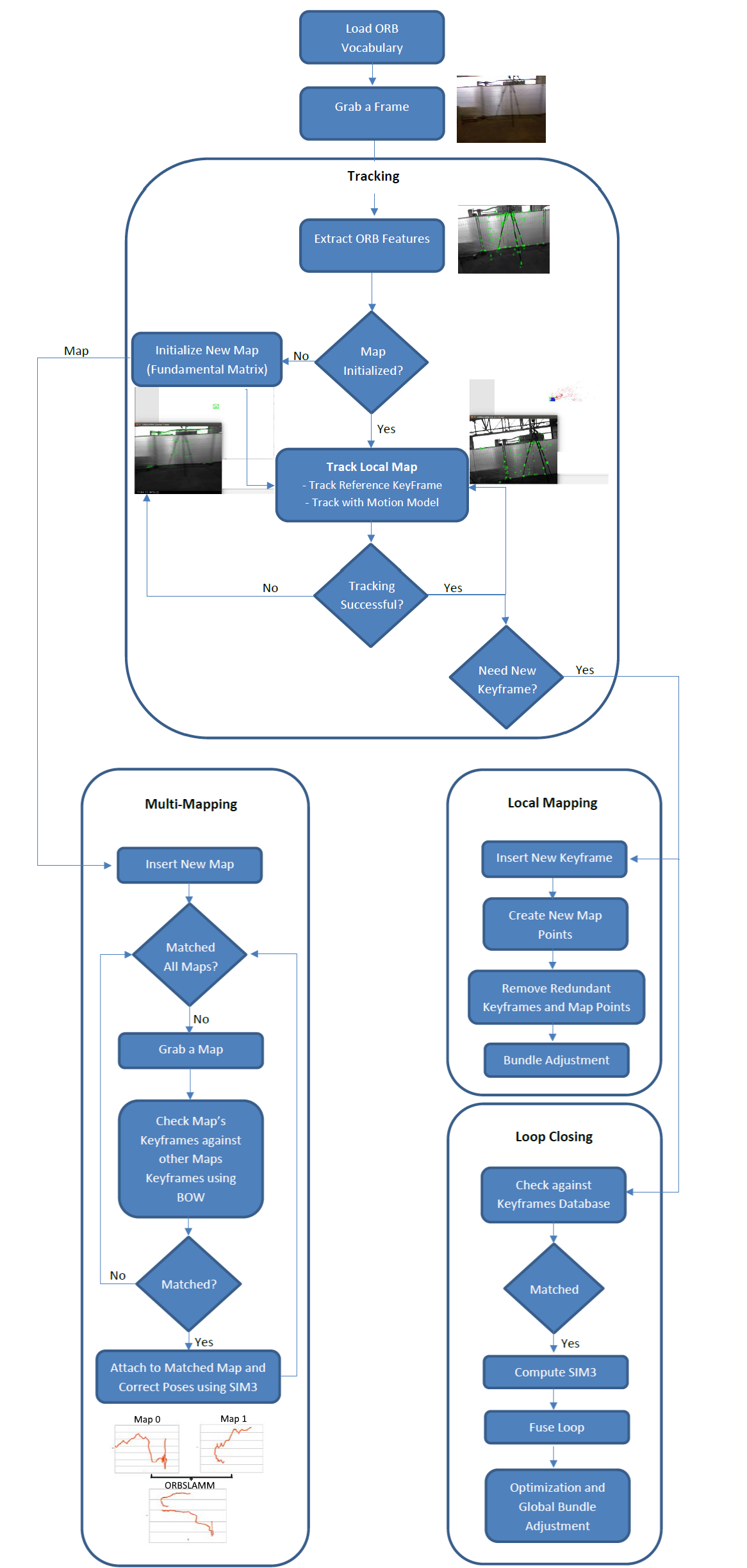

Supplement: S1 Fig — (TIF) [file pone.0195878.s004.tif]

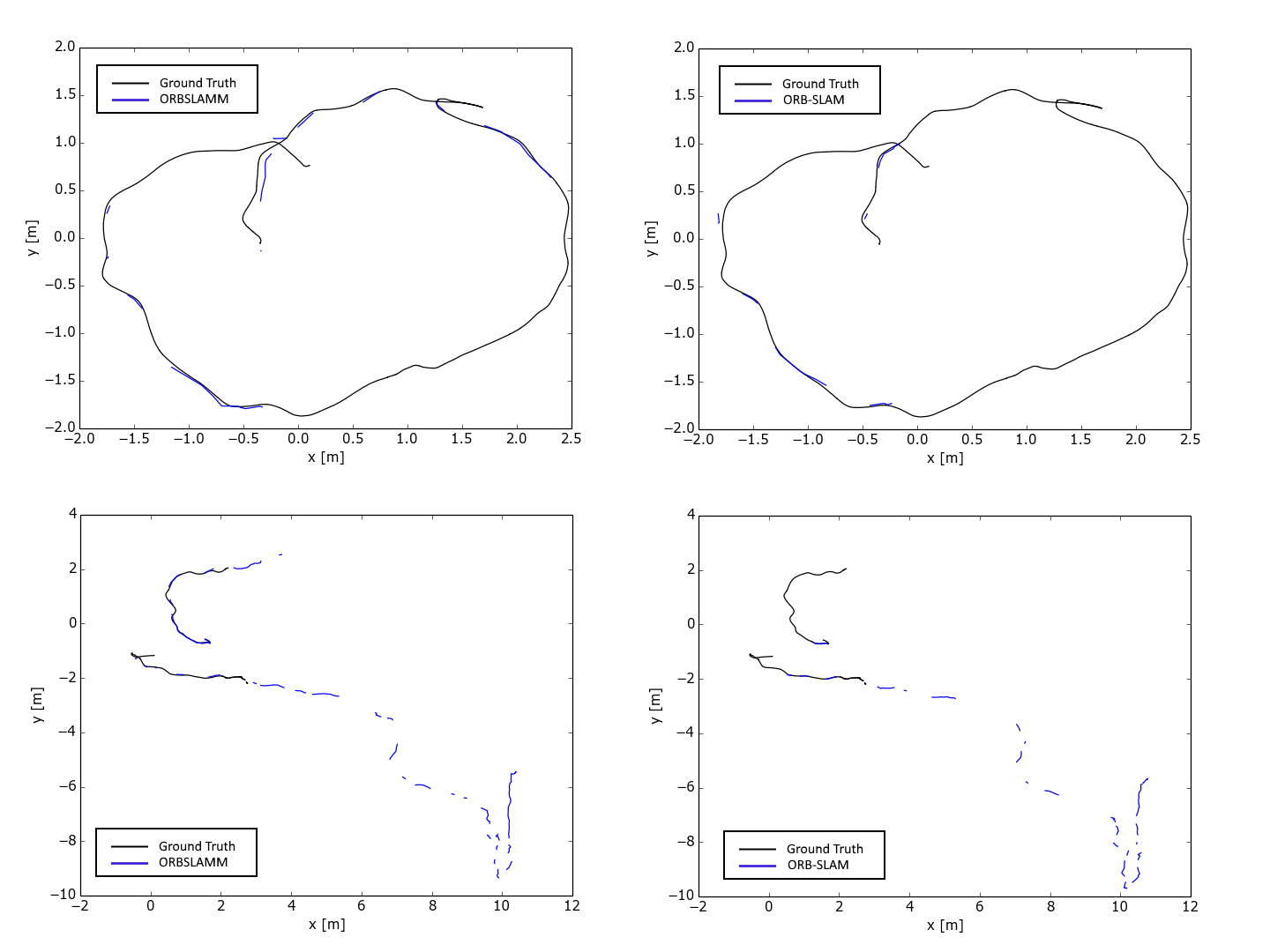

Supplement: S2 Fig — Comparison against ground-truth after alignment and scale correction. The upper row is sequence fr2_360_kidnap and the lower row is sequence fr2_large_with_loop. The left column is for ORBSLAMM while the right column is for ORB-SLAM. (TIF) [file pone.0195878.s005.tif]

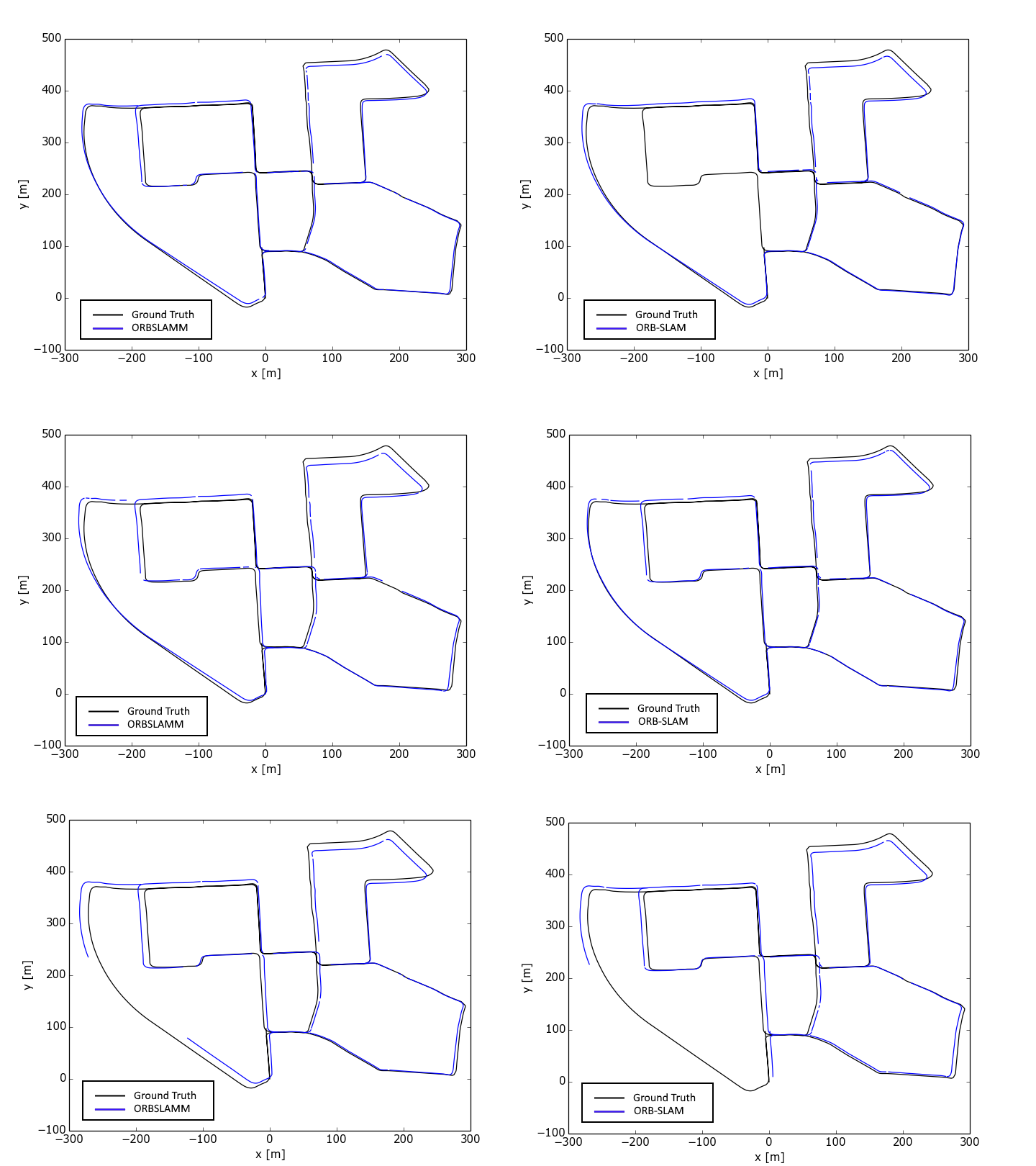

Supplement: S3 Fig — The first row is for error at 10%, the second row is for error at 50% and the third row is for error at 90%. The left column is for ORBSLAMM and the right column is for ORB-SLAM. (TIF) [file pone.0195878.s006.tif]

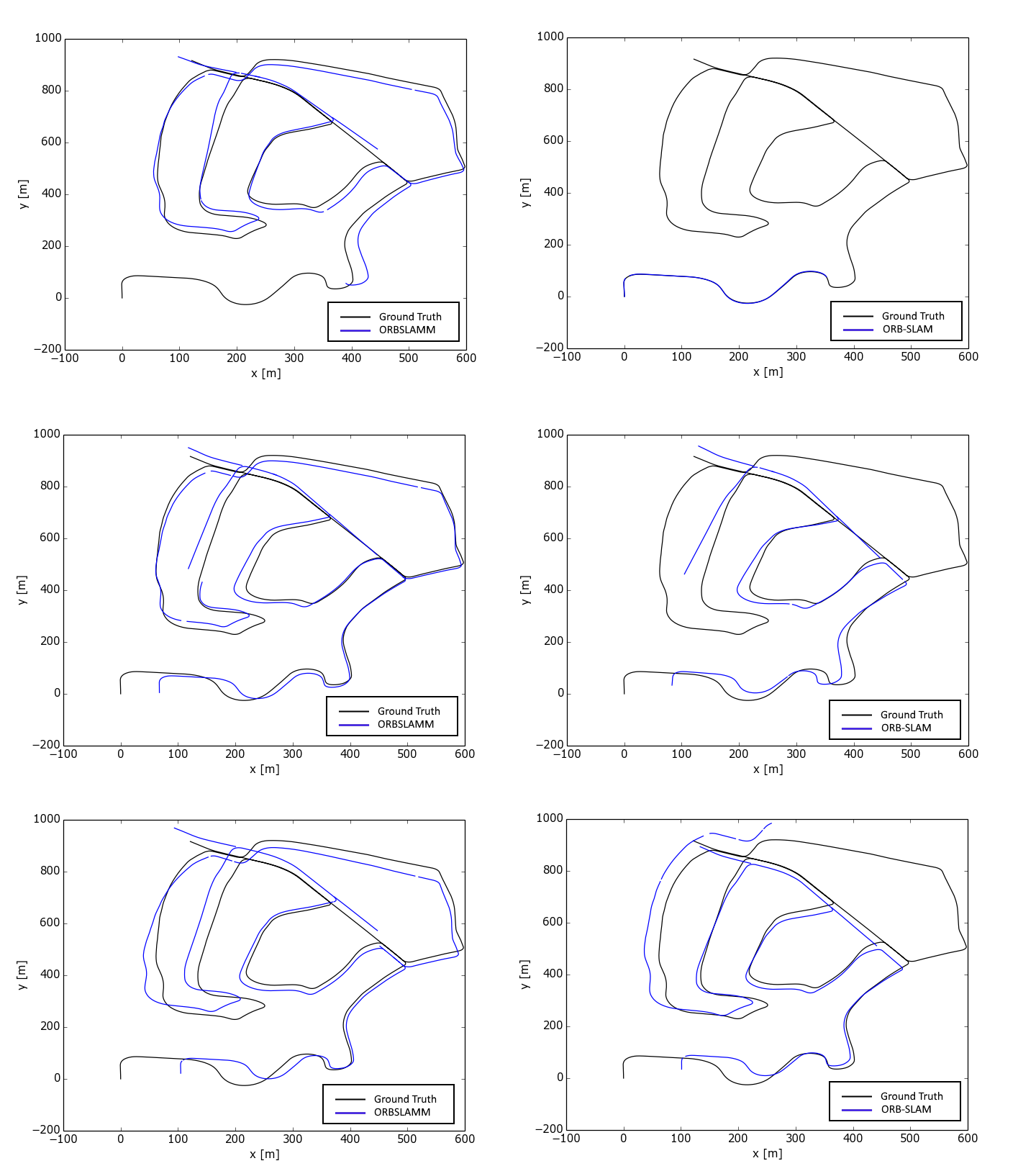

Supplement: S4 Fig — The first row is for error at 10%, the second row is for error at 50% and the third row is for error at 90%. The left column is for ORBSLAMM and the right column is for ORB-SLAM. (TIF) [file pone.0195878.s007.tif]

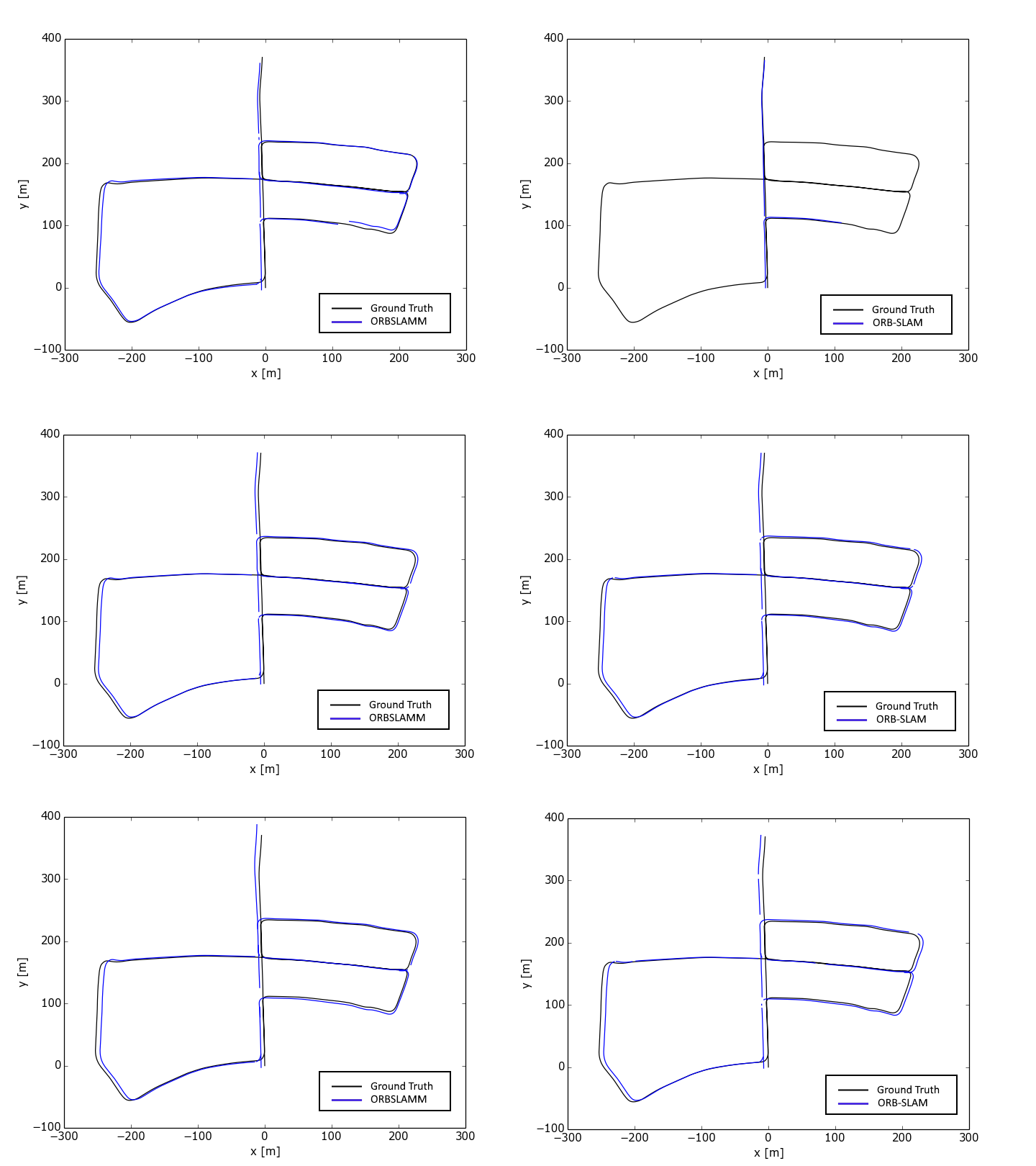

Supplement: S5 Fig — The first row is for error at 10%, the second row is for error at 50% and the third row is for error at 90%. The left column is for ORBSLAMM and the right column is for ORB-SLAM. (TIF) [file pone.0195878.s008.tif]

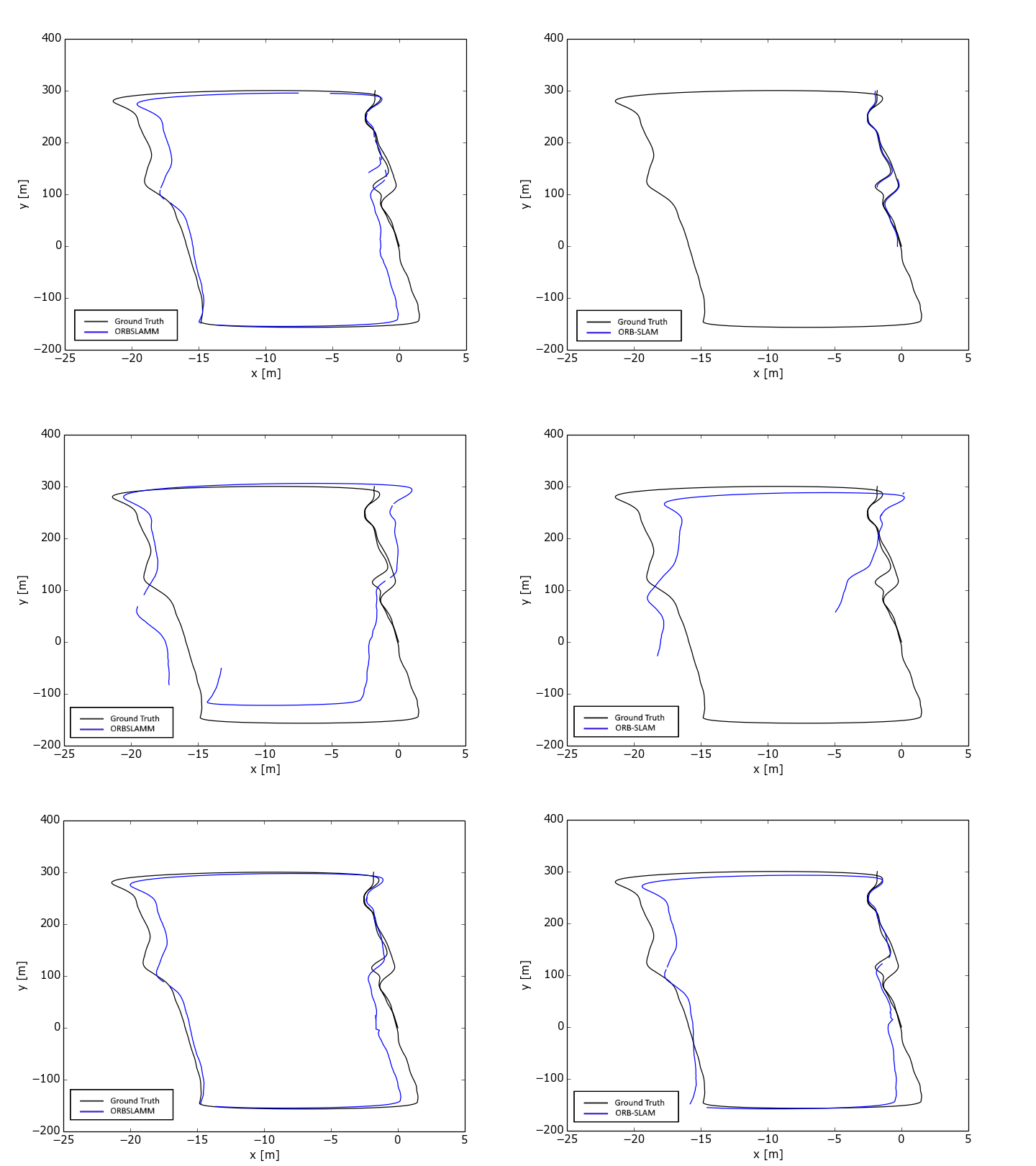

Supplement: S6 Fig — The first row is for error at 10%, the second row is for error at 50% and the third row is for error at 90%. The left column is for ORBSLAMM and the right column is for ORB-SLAM. (TIF) [file pone.0195878.s009.tif]

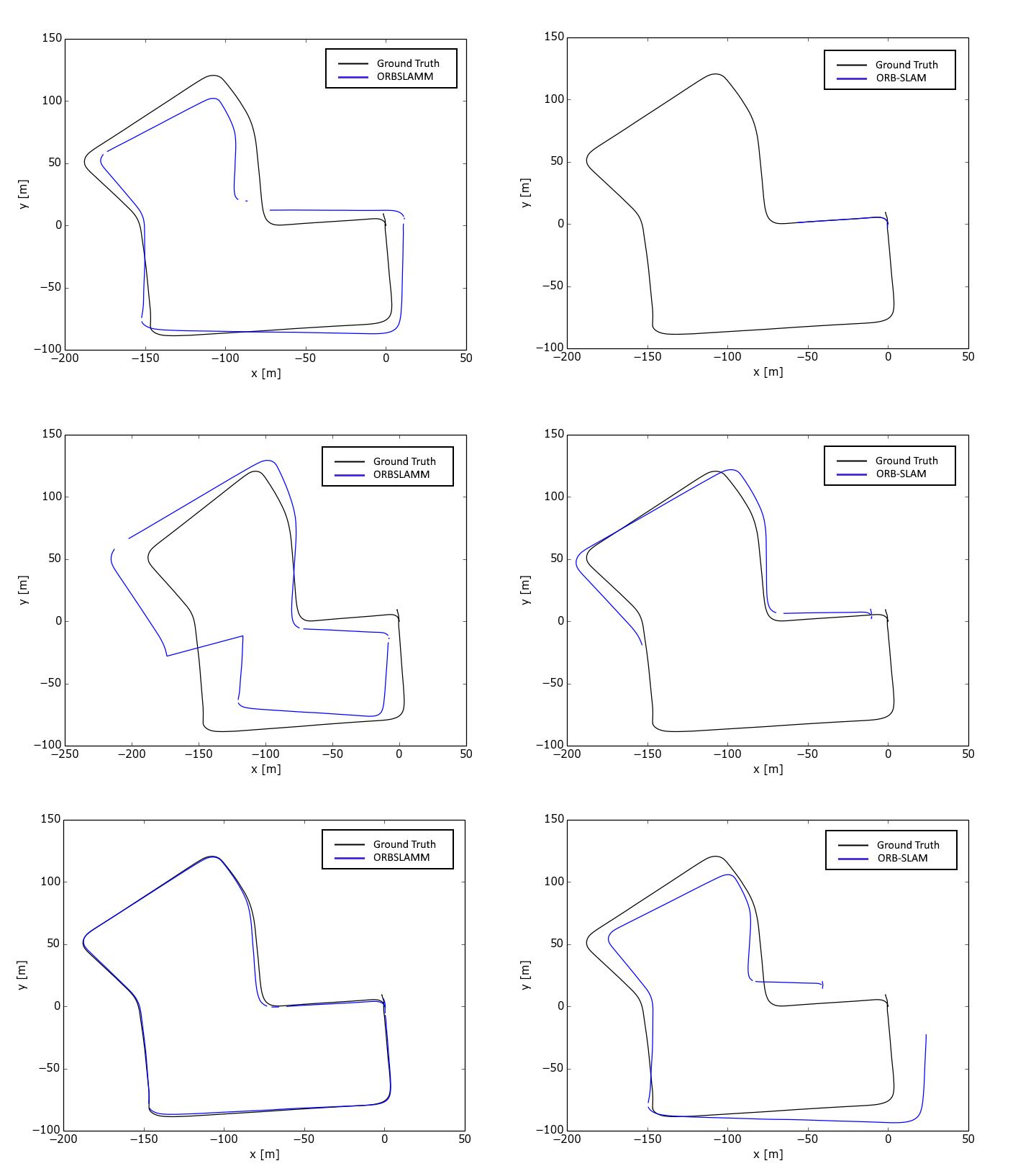

Supplement: S7 Fig — The first row is for error at 10%, the second row is for error at 50% and the third row is for error at 90%. The left column is for ORBSLAMM and the right column is for ORB-SLAM. (TIF) [file pone.0195878.s010.tif]

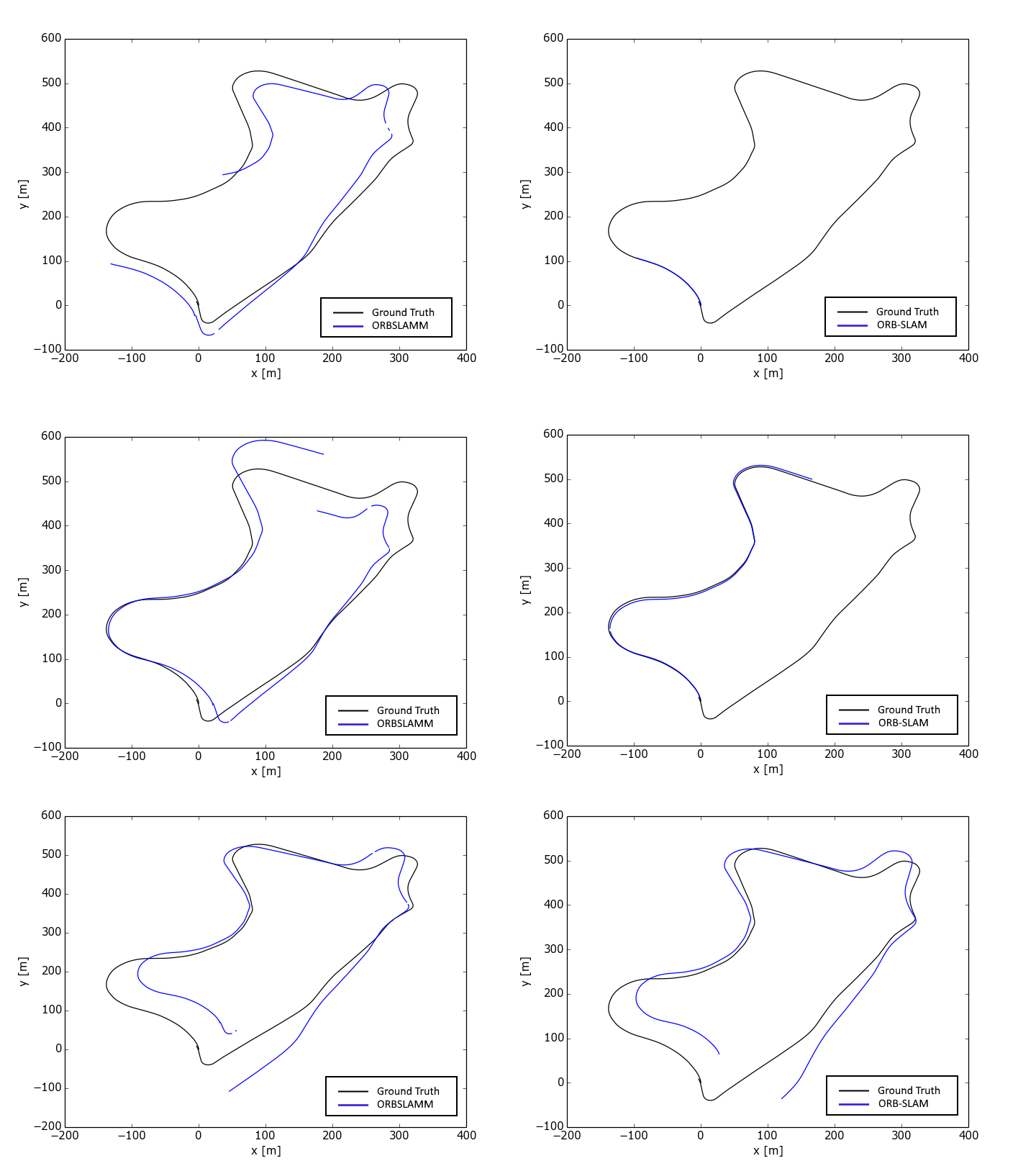

Supplement: S8 Fig — The first row is for error at 10%, the second row is for error at 50% and the third row is for error at 90%. The left column is for ORBSLAMM and the right column is for ORB-SLAM. (TIF) [file pone.0195878.s011.tif]
